# Trajectories through semantic spaces in schizophrenia and the relationship to ripple bursts

**DOI:** 10.1073/pnas.2305290120

**Published:** 2023-10-10

**Authors:** Matthew M. Nour, Daniel C. McNamee, Yunzhe Liu, Raymond J. Dolan

**Affiliations:** ^a^Department of Psychiatry, University of Oxford, Oxford OX3 7JX, United Kingdom; ^b^Max Planck University College London Centre for Computational Psychiatry and Ageing Research, London WC1B 5EH, United Kingdom; ^c^Champalimaud Research, Centre for the Unknown, 1400-038 Lisbon, Portugal; ^d^State Key Laboratory of Cognitive Neuroscience and Learning, IDG/McGovern Institute for Brain Research, Beijing Normal University, Beijing 100875, China; ^e^Chinese Institute for Brain Research, Beijing 102206, China; ^f^Wellcome Centre for Human Neuroimaging, University College London, London WC1N 3AR, United Kingdom

**Keywords:** cognitive map, psychosis, hippocampal replay, sharp wave ripple, natural language processing

## Abstract

Human cognition is underpinned by structured internal representations that encode relationships between entities in the world (cognitive maps). Clinical features of schizophrenia—from thought disorder to delusions—are proposed to reflect disorganization in such conceptual representations. Schizophrenia is also linked to abnormalities in neural processes that support cognitive map representations, including hippocampal replay and high-frequency ripple oscillations. Here, we report a computational assay of semantically guided conceptual sampling and exploit this to test a hypothesis that people with schizophrenia (PScz) exhibit abnormalities in semantically guided cognition that relate to hippocampal replay and ripples. Fifty-two participants [26 PScz (13 unmedicated) and 26 age-, gender-, and intelligence quotient (IQ)-matched nonclinical controls] completed a category- and letter-verbal fluency task, followed by a magnetoencephalography (MEG) scan involving a separate sequence-learning task. We used a pretrained word embedding model of semantic similarity, coupled to a computational model of word selection, to quantify the degree to which each participant’s verbal behavior was guided by semantic similarity. Using MEG, we indexed neural replay and ripple power in a post-task rest session. Across all participants, word selection was strongly influenced by semantic similarity. The strength of this influence showed sensitivity to task demands (category > letter fluency) and predicted performance. In line with our hypothesis, the influence of semantic similarity on behavior was reduced in schizophrenia relative to controls, predicted negative psychotic symptoms, and correlated with an MEG signature of hippocampal ripple power (but not replay). The findings bridge a gap between phenomenological and neurocomputational accounts of schizophrenia.

## Significance

Schizophrenia is a debilitating neuropsychiatric disorder whose core clinical features are thought to reflect abnormalities in internal conceptual representations (“cognitive maps”). The current work provides a language-based computational assay of conceptual disorganization in schizophrenia and relates this to neural signatures of cognitive map representation measured using magnetoencephalography (MEG). At a behavioral level, patients with schizophrenia showed reduced semantically guided word sampling during a verbal fluency task (a marker of “looser” conceptual organization). At a neural level, between-participant variance in this effect correlated with the strength of an MEG signature of hippocampal ripple power (measured in a separate task), known to be involved in cognitive map stabilization. These findings shed light on the neural basis of semantic representation in schizophrenia.

Schizophrenia is a common and debilitating neuropsychiatric disorder. The condition has a lifetime prevalence of 0.7% and remains a leading cause of years lived with disability (1–3). Core clinical features include conceptual disorganization, difficulties in abstract reasoning, and reduced language coherence (i.e., “formal thought disorder”) (4, 5), which together predict poor social functioning (6). A longstanding notion is that these features reflect an abnormality in association-guided cognition (7, 8), where associations reflect relationships between memories, concepts, or objects in the world (“relational knowledge”) (9–11).

The question of how abstract relational knowledge is represented in the brain has gained renewed prominence in cognitive neuroscience, with convergent evidence identifying a role for hippocampal–entorhinal (HEC) cortex. This is exemplified by rodent findings showing hippocampal place and grid cells encode relationships between spatial locations during navigation (12), as well as during rest-period neural “replay” (i.e., sequential place cell reactivation that reflects spatial proximity, expressed during high-frequency hippocampal “ripple” oscillations) (13). Of relevance to psychopathology are findings that HEC also encodes associations between states in more abstract domains (e.g., associations between pictures in a task, or semantic relationships between words) (14–19), indicating that HEC supports a more domain-general “cognitive mapping” function (14, 15) spanning conceptual and semantic “spaces.” Thus, when people perform word recall tasks, HEC theta power covaries with “semantic proximity” between sequentially recalled words (20), while the order of word selection in verbal fluency tasks can be captured using models initially devised to describe behavior in foraging animals (akin to “mental navigation” in a semantic space) (21, 22).

Given a proposed function of HEC in domain-general cognitive mapping, we hypothesized that neural replay and ripple power would relate to patterns of spontaneous word selection during verbal fluency. The underlying intuition is that the stream of consciousness can be construed as a trajectory through a “semantic space” (23, 24), where the sequential sampling of concepts is more or less constrained by some notion of semantic “proximity.” Speculatively, abnormalities in this neural process could also relate to disrupted semantic sampling patterns in patients (a candidate behavioral assay of formal thought disorder). Intriguingly, schizophrenia is associated with abnormalities in both hippocampal replay and ripple oscillations, both in genetic mouse models (25–27) and human subjects (11), but the relationship to conceptual sampling and language metrics is unknown.

Here, we use computational modeling of verbal fluency data to derive measures of semantically structured conceptual sampling in patients with a diagnosis of schizophrenia (PScz) and intelligence quotient (IQ)-matched control participants. We leverage advances in Natural Language Processing (NLP) machine learning tools to provide a quantification of semantic associations between words, an approach increasingly applied to the study of natural language and semantic processing both in psychiatry (9, 28–32) and cognitive neuroscience (20, 33–39). We then relate model-derived measures of semantic sampling to magnetoencephalography (MEG) signatures of neural replay and replay-associated ripple oscillations in the same participants, measured in a rest session following a separate task. Our hypothesis was that schizophrenia is linked to behavioral impairments in semantically guided conceptual sampling and that these relate to neural signatures of structured memory reactivations during rest (replay and ripples) considered to emanate from HEC.

## Results

### Trajectories through Semantic Space.

**Fig. 1.**
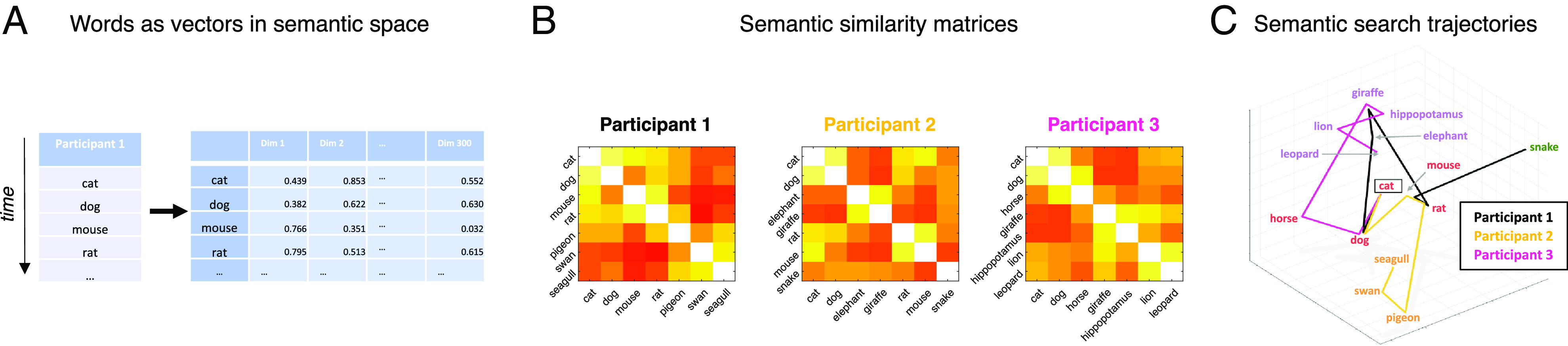
Item responses represented as vectors in semantic space. (*A*) In the category task participants named as many animals as they could in 5 min. Words were embedded within a common 300-dimensional semantic space using a pretrained word-embedding model (40). (*B*) Semantic proximity (association) between any two response items was defined as the cosine similarity between item vectors (color axis 0 to 0.8). (*C*) Initial word lists for 3 PScz visualized as trajectories through semantic space [3-dimensional projection derived from Uniform Manifold Approximation and Projection (UMAP) algorithm (41) applied to [item, 300] embedding matrix using cosine distance in ambient space. Item color from data-driven community assignment (see Fig. 4)].

A total of 52 participants [26 PScz (13 not taking any psychiatric medication) and 26 control participants, see *SI Appendix*, Table S1] completed two verbal fluency tasks, wherein they were asked to name as many words as they could either “belonging to the category ‘animals’” (category fluency) or “starting with the letter ‘p’” (letter fluency) in 5 min. For each task, we used a fastText (Facebook AI Research) pretrained NLP word embedding model (40) to quantify the semantic association (proximity) between each pair of response items in terms of cosine similarity (1-cosine distance) (Fig. 1).

**Fig. 2.**
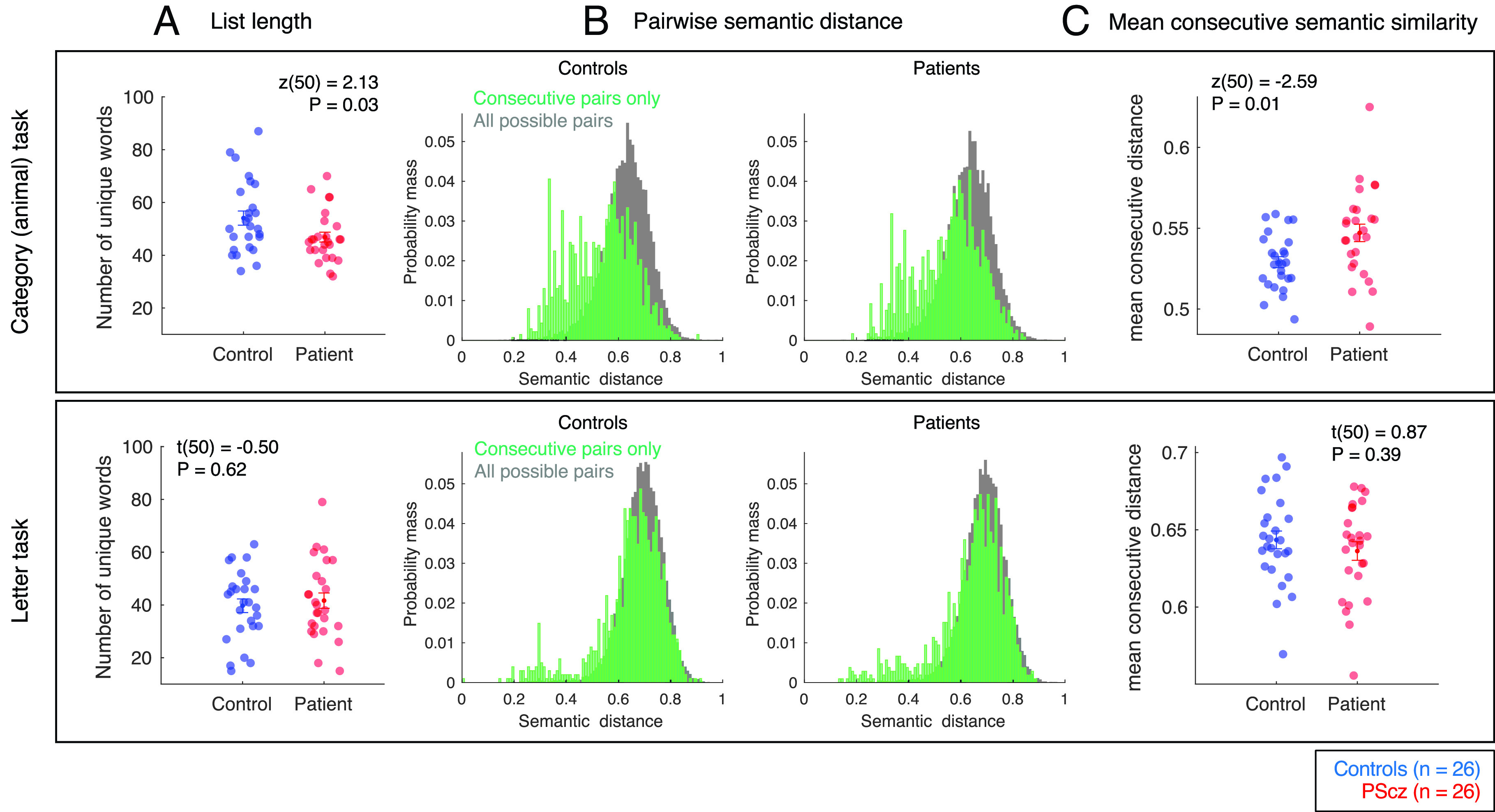
Category (“animal”) and letter (“p” words) tasks: list length and pairwise semantic distances. (*A*) Mean ± SEM number of unique valid words generated in 5 min. See *SI Appendix* for further results. (*B*) Distribution of semantic distances for consecutively emitted word pairs (green) vs. distances between all unique words (gray). Distribution corresponds to concatenated lists over all control participants (*Left*) and PScz (*Right*). See *SI Appendix* for further results. (*C*) Mean ± SEM semantic distance between consecutive words (unique words only). Sample: n = 26 controls, n = 26 PScz. *Top:* category fluency. *Bottom:* letter fluency. Group comparison statistics from two sample *t* tests (t) and Wilcoxon rank sum tests (z), two-tailed.

In the “category” task, control participants exhibited lower mean semantic distances (i.e., increased semantic similarity) between consecutively generated items compared to PScz [control: mean semantic distance = 0.53 ± 0.003 (SEM), PScz: mean semantic distance = 0.55 ± 0.005, z(50) = −2.59, *P* = 0.01, Wilcoxon rank sum test, two-tailed]. This group difference was not present for the “letter” task [control: mean semantic distance = 0.643 ± 0.006, PScz: mean semantic distance = 0.636 ± 0.006, t(50) = 0.87, *P* = 0.39, two-sample *t* test, two-tailed, Fig. 2]. A group * task ANOVA confirmed a significant interaction effect on mean consecutive semantic distance [F(50, 1) = 5.44, *P* = 0.024], in addition to a significant main effect of task [F(50, 1) = 456.5, *P* < 0.001], with no main effect of group [F(50, 1) = 0.20, *P* = 0.66].

**Fig. 3.**
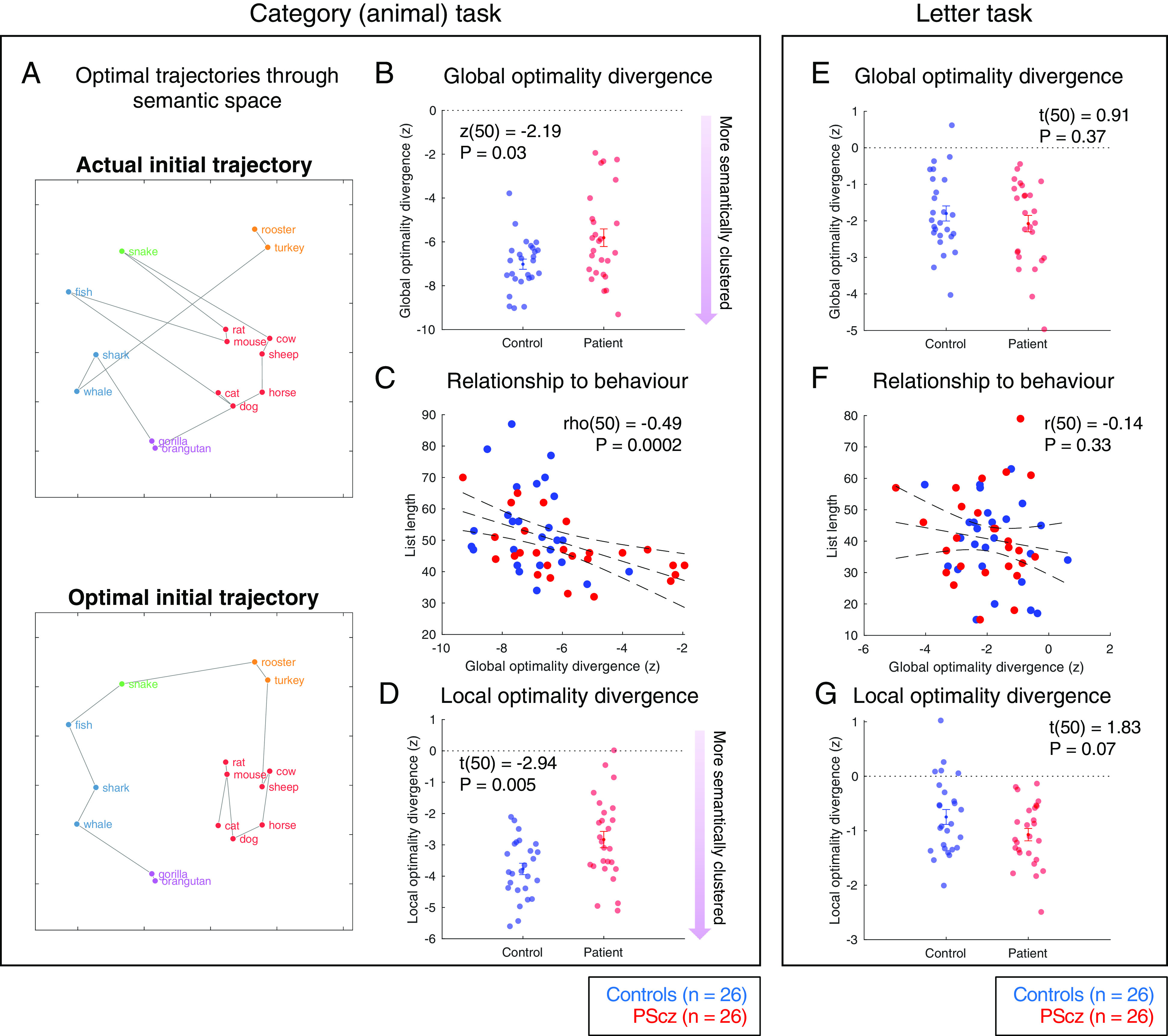
Semantic path optimality analysis. (*A*). First 15 words in the observed (*Top*) and optimal (*Bottom*) semantic search path of one example PScz (first word: “cat”). Optimal paths denote the shortest Hamiltonian path in original embedding space. 2-dimensional UMAP visualization (41) using cosine distance in ambient space, intended for illustration purposes only (i.e., optimal paths defined using cosine distance in ambient 300 dimensional word embedding space). Item color from data-driven community assignment (Fig. 4). (*B*) Global optimality divergence (distance_observed_-distance_optimal_) expressed as a z-score against participant-specific list shuffles (negative values indicate increasing deviation from a random word selection process, i.e., more semantically clustered behavior). (*C*) Global optimality divergence (in *B*) predicts task performance (number of items emitted). Spearman’s correlation, two-tailed. (*D*) Local optimality divergence, quantifying the degree to which observed and optimal paths align at the level of item-item transitions (edges). For a given item list, we assigned every consecutive item–item transition in the observed path a score, corresponding to the number of edges separating the relevant item pair in the optimal path. Local optimality divergence is the sum of these scores across all consecutive item pairs in the observed path, expressed as a z-score as in (*B*). (*E*) As (*B*) but using data from the letter task. (*F*) As (*C*) but using data from the letter task. Pearson’s correlation, two-tailed. (*G*) As (*D*) but using data from the letter task. Group comparisons (*B*, *D*, *E* and *G*) expressed as mean ± SEM. Group comparison statistics from two sample *t* tests (t) and Wilcoxon rank sum tests (z), two-tailed. Error bars on linear trend lines (*C* and *F*) reflect 95% CI of the linear regression slope. Length of sliding analysis window (*SI Appendix*) equals the task-specific minimum list length across all participants [for category task = 32, for letter task = 15 (for similar results with window length = 32 in letter task, see *SI Appendix*, Fig. S2)]. Sample: n = 26 controls, n = 26 PScz.

To better characterize the influence of semantic association strength on response item selection, for each participant, we next defined measures of path optimality by comparing the observed item sequence to an “optimal” sequence. This optimal sequence was defined as the sequence which visits each item exactly once in an order that minimizes the total semantic distance traveled (i.e., the shortest Hamiltonian path defined on a complete graph where the weight of an undirected edge connecting any two items corresponds to their cosine similarity, and where this path is derived using a modified “Travelling Salesman” algorithm. Fig. 3*A*). We defined “global optimality divergence” as the difference between the observed and optimal total path distance. We expressed this metric as a z-score with respect to participant-specific shuffled item lists, thus controlling for variation in item sets between participants (see *SI Appendix* for details).

In the category task, global optimality divergence was lower for control participants compared to PScz [control: mean −7.02 ± 0.23, PScz: mean = −5.81 ± 0.40, z(50) = −2.19, *P* = 0.03, Wilcoxon rank sum test, two-tailed, Fig. 3*B*, negative values reflect increasingly optimal word selection processes and 0 indicates random word selection]. This measure correlated with task performance [total number of words generated, rho(50) = −0.49, *P* = 0.002, Spearman’s rank correlation, Fig. 3*C*], with the observed association being preserved even when controlling for an effect of group on list length in a multiple regression analysis [list length ~ group * optimality divergence multiple regression: $\beta_{\mathrm{optimality\_divergence}}$ = −2.71 ± 1.10, t(48) = −2.47, *P* = 0.017. $\beta_{\mathrm{group}}$ = 0.41 ± 15.1, t(48) = 0.27, *P* = 0.98. $\beta_{\mathrm{group}*\mathrm{optimality}\_\mathrm{divergence}}$ = −0.55 ± 2.19, t(48) = −0.25, *P* = 0.80]. All of these results were robust when considering only the first 32 items from each participant’s list (the length of the shortest list over participants), demonstrating that effects are not explained by differences in list length between participants.

We also defined a related “local optimality divergence” metric as the degree to which optimal and observed trajectories are aligned at the level of individual item–item transitions (see *SI Appendix* for details). Using this measure, control participants also exhibited greater similarity to optimal path trajectories than PScz [controls: mean = −3.77 ± 0.18, PScz: mean = −2.83 ± 0.26, t(50) = −2.94, *P* = 0.005, two sample *t* test, two-tailed, Fig. 3*D*].

To test the sensitivity of these findings to task context, we repeated this analysis on data from the letter fluency task (“name all words beginning with ‘p’”). This task does not require explicit consideration of semantic information and performance is reported to be less affected in psychosis (42–44). In keeping with these prior findings, we found no group differences in path optimality measures nor any correlation with overall task performance (Fig. 3 *E–G*).

### Trajectories through Semantic Communities.

**Fig. 4.**
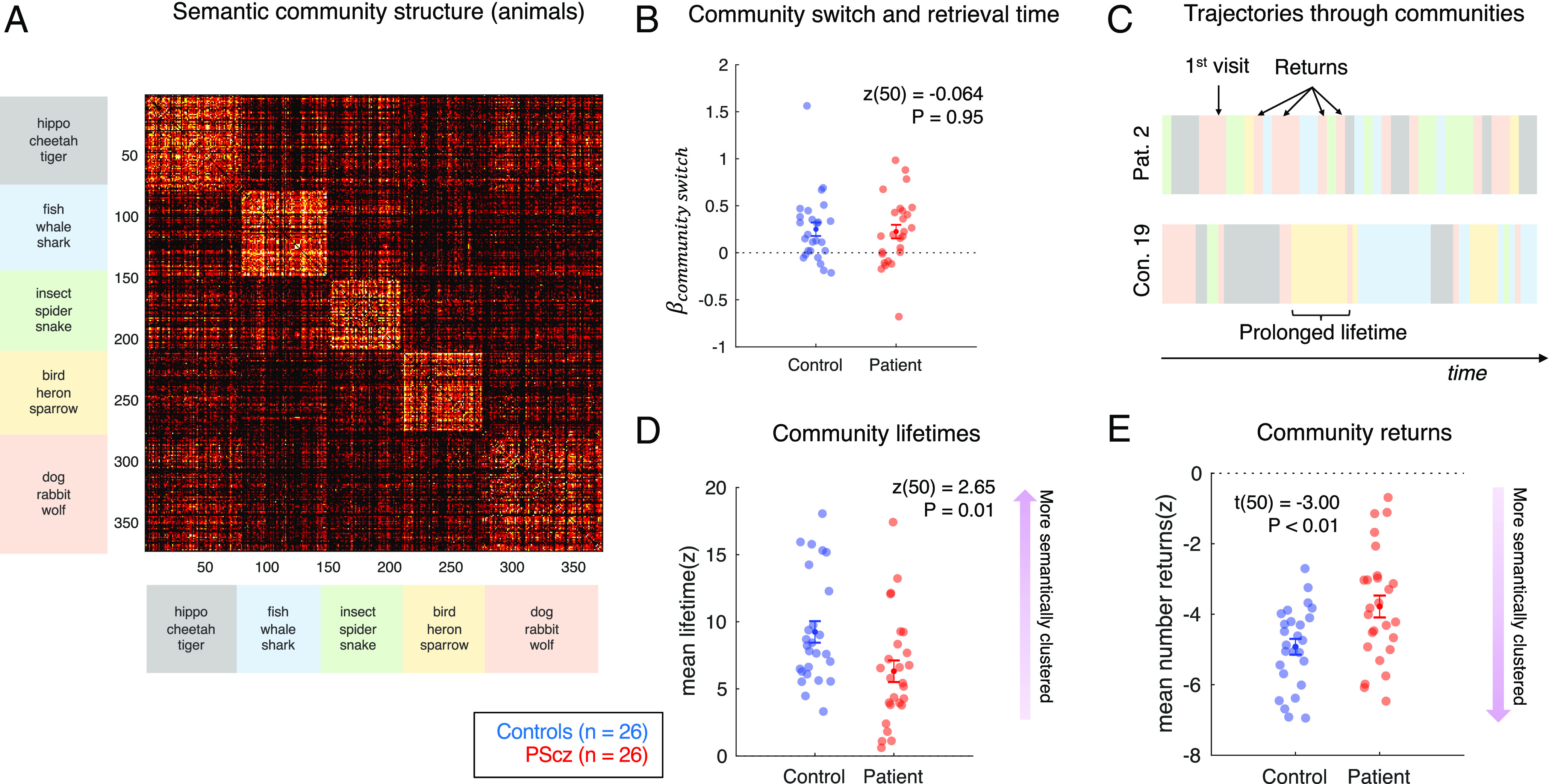
Trajectories through semantic communities in category task. (*A*) Semantic similarity (1-cosine distance) matrix of all unique response items, ordered by community assignment from Louvain agglomerative clustering algorithm (45). Communities labeled with the first three animal words exhibiting the highest cosine similarity to the cluster center of mass in the fastText vocabulary. Color axis 0.3 to 0.6. (*B*) For each participant, we regressed (log) retrieval time for each item (RT, duration between consecutive items) onto a design matrix comprising 1) pairwise semantic distance between current item and previous item (demeaned), 2) presence of a “community switch” [binary variable, as defined by community partition in (*A*)], 3) item response number (as proportion of list length), and 4) a constant term. Figure shows mean ± SEM of the regression coefficient associated with community switches, showing a significant effect greater than 0 in both controls and PScz (i.e., RT slowing with community switch), with no significant group difference (Wilcoxon rank sum test, two-tailed). (*C*) Two participant trajectories, where items are color coded according to community membership in (*A*) illustrating key “community trajectory” features. (*D*) Community “lifetimes” (mean ± SEM number of consecutive words that belong to the same community in a given item list). (*E*) Community “returns” (mean ± SEM number of times a participant’s item list revisits a semantic community after exiting it for the first time). Effects in (*D*) and (*E*) expressed as a z-score with respect to participant-specific null distribution of effects from shuffled lists, and averaged over all consecutive sliding analysis windows, as in item-level trajectory analysis. Group comparison statistics from two sample *t* tests (t) and Wilcoxon rank sum tests (z), two-tailed. Sample: controls n = 26, PScz n = 26. Community partition performed using the Louvain algorithm (45) with resolution parameter set to 1 (see *SI Appendix*, Fig. S5 for details and resolution parameter sensitivity analysis).

Given the group differences seen in the category task, we next examined the degree to which behavior was sensitive to mesoscale structure of the semantic space (“animal space”), which we partitioned into “semantic communities” using a data-driven agglomerative clustering procedure (Fig. 4*A*, see *SI Appendix* for details). While category fluency behavior in both PScz and control participants was sensitive to the identified community partition, this sensitivity was again reduced in PScz. Specifically, compared to controls, PScz lists displayed shorter uninterrupted stretches of items within the same community [mean community “lifetime” in controls expressed as z-score with respect to participant shuffled lists = 9.25 ± 0.80, PScz = 6.32 ± 0.80, z(50) = 2.65, *P* = 0.008, Wilcoxon rank sum test, two-tailed, Fig. 4*D*] and an increased propensity to return to a community after it had already been visited [number of returns in controls expressed as z-score with respect to participant shuffled lists = −4.92 ± 0.22, PScz = −3.78 ± 0.31, t(50) = −3.00, *P* = 0.004, two sample *t* test, two-tailed, Fig. 4*E*].

### Trajectories through Orthographic Space.

Non-semantic word relationships have also been shown to affect performance in learning, memory, and language tasks (46, 47). We therefore conducted an analogous trajectory analysis using a non-semantic distance metric sensitive to the letter-by-letter similarity between words [orthographic or “Levenshtein” distance (48)]. As expected, orthographic distances between consecutive items were lower in the letter task compared to the category task, and the degree of orthographic clustering (global optimality divergence) predicted performance in the letter task alone. However, we found no group differences in optimality divergence defined using orthographic distance in either task (see *SI Appendix*, Fig. S3).

### Modeling Semantic Retrieval as Local Search.

**Fig. 5.**
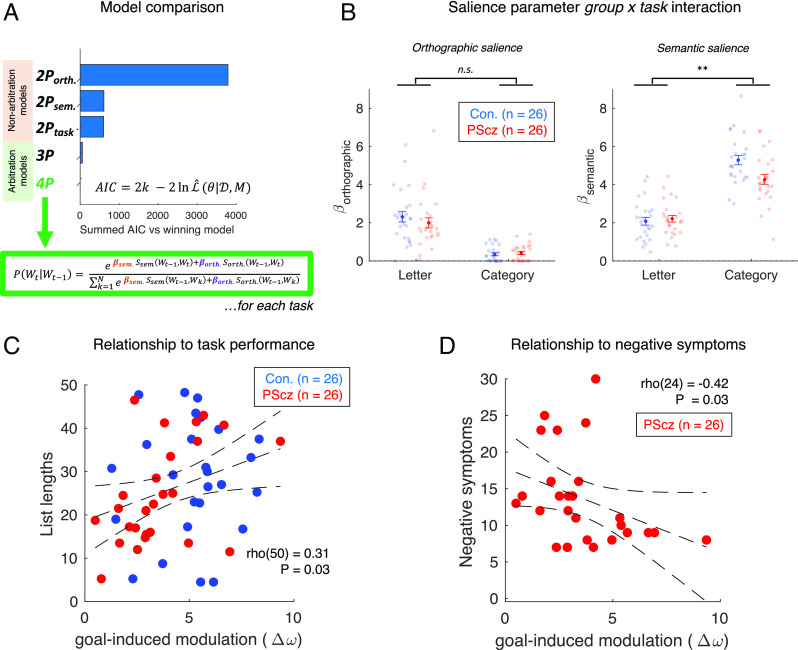
Modeling task behavior as a local search in semantic space. (*A*) Model comparison, summed AIC over all participants (see *SI Appendix* for model specifications). The winning (4 parameter) model asserts that the probability of observing the *t-*th emitted word ( $W_{t}$ ) is a softmax function of both the semantic and orthographic association strength between $W_{t}$ and the previous emitted word (i.e., $S\left(W_{t-1},W_{t}\right)$ ), which are multiplicatively scaled by separate free salience parameters β . Data from letter and category tasks modeled separately (i.e., this model may be construed as two independent 2-parameter models, one for each task, as shown in equation). (*B*) Group * task ANOVA for orthographic (*Left*) and semantic (*Right*) salience fitted parameters from the winning model (in *A*). Significant interaction effect for semantic saliencies (** denotes *P* < 0.01), indicating that control participants exhibit an increased boosting of semantic salience in category vs. letter task, compared to PScz. (*C*) Across participants, we find a positive correlation between goal-induced semantic modulation ( Δω, a contrast of fitted model parameters quantifying how relative sensitivity to semantic associations increases in line with task context) and performance (mean list length across both tasks, where the length of each list is first expressed as a task-specific rank across participants to account for differences in the distribution of list lengths between tasks). (*D*) The relationship between negative symptoms of schizophrenia (49) and Δω . For (*C*) and (*D*), correlation coefficients from Spearman’s correlation. Dashed trend lines represent 95% CI on linear line of best fit. Sample: n = 26 controls, n = 26 PScz.

To ascertain the contribution of semantic association on word selection with finer granularity we next turned to computational modeling of behavior and formally tested for a group * task interaction effect in semantic association strength. In the models considered, for response item t , a single word is selected from the set of N unique words generated across all participants, and the probability of emitting any given word from this set is a function of the semantic and/or orthographic proximity to response item t-1 , multiplicatively scaled by a salience ( β ) parameter. The winning model (identified in formal model comparison) allowed for an arbitration between semantic and orthographic association strength in the word selection process and modeled this trade-off independently in category and letter tasks for each participant (i.e., four free parameters: a pair of semantic and orthographic salience parameters for category [ $\beta_{\mathrm{sem}.\mathrm{CAT}}$ & $\beta_{\mathrm{orth}.\mathrm{CAT}}$ ] and letter tasks [ $\beta_{\mathrm{sem}.\mathrm{LETT}}$ & $\beta_{\mathrm{orth}.\mathrm{LETT}}$ ] separately (Fig. 5*A*). (See *SI Appendix* for mathematical details, model comparison, parameter recovery and fitted parameter correlations.)

The fitted model parameters recapitulated the model-agnostic results (above), revealing main effects of task condition on the relative weighting between semantic and orthographic similarities (semantic > orthographic for category task, and orthographic > semantic for letter task). Crucially, in the category task, PScz and control participants showed a significant difference in semantic salience alone [ $\beta_{\mathrm{sem}.\mathrm{CAT}}:$ t(50) = 2.86, *P* = 0.01, two sample *t* test, two-tailed], with no group difference for orthographic salience [ $\beta_{\mathrm{orth}.\mathrm{CAT}}:$ z(50) = −0.32, *P* = 0.75, Wilcoxon rank sum test, two-tailed]. Groups did not differ on either salience parameter in the letter task [ $\beta_{\mathrm{sem}.\mathrm{LETT}}:$ t(50) = −0.50, *P* = 0.62 and $\beta_{\mathrm{orth}.\mathrm{LETT}}:$ z(50) = 1.02, *P* = 0.31]. Accordingly, group * task ANOVA on the semantic salience parameter confirmed a significant interaction ( $\beta_{\mathrm{sem}.}$ : group * task *P* = 0.005), with no such effect for the orthographic salience parameter [ $\beta_{\mathrm{orth}.}$ : group * task interaction *P* = 0.34] (Fig. 5*B*).

As the winning model captured a trade-off between semantic and orthographic saliencies, this enabled us to ascertain the degree to which semantic salience exceeds orthographic salience for each condition ( $\omega =\beta_{\mathrm{sem}.}-\beta_{\mathrm{orth}.}$ ), as well as quantify the degree to which this difference metric changes as a function of task context ( $\Delta \omega =\omega_{\mathrm{CAT}}-\omega_{\mathrm{LETT}}$ , which we term “goal-induced semantic modulation”). Thus, Δω captures a tendency to modulate the associative information guiding memory search in line with task demands. Δω was significantly correlated with both “global” and “local” semantic optimality divergence measures in the category task [global: rho(50) = −0.56, *P* < 0.001; local: rho(50) = −0.60, *P* < 0.001, Spearman’s correlation].

As expected, goal-induced semantic modulation ( Δω ) differed between groups [controls: mean Δω = 5.17 ± 0.37, PScz: mean Δω = 3.63 ± 0.40, t(50) = 2.82, *P* = 0.01, two sample *t* test, two-tailed], and predicted performance [rho(50) = 0.31, *P* = 0.03, Spearman’s correlation, Fig. 5*C*], with no group difference in this latter relationship [list length ~ group* Δω multiple regression: $\beta_{\Delta \omega }$ = 1.62 ± 0.87, t(48) = 1.86, *P* = 0.07. $\beta_{\mathrm{group}}$ = 11.2 ± 8.56, t(48) = 1.31, *P* = 0.20. $\beta_{\mathrm{group}*\Delta \omega }$ = −2.32 ± 1.74, t(48) = −1.33, *P* = 0.19].

### Relationship to Symptoms and Cognitive Variables.

We found an inverse relationship between total negative symptom score (49) and goal-induced semantic modulation [ Δω , rho(24) = −0.42, *P* = 0.03, Spearman’s correlation, Fig. 5*D*]. This was driven by symptom items that relate to “negative thought disorder” (5) (“N5: difficulty in abstract thinking,” rho = −0.47, *P* = 0.015, “N6: lack of spontaneity and flow of conversation,” rho = −0.43, *P* = 0.028), which capture difficulties with proverb understanding, semantic similarity assessments, and abstract vs. concrete modes of reasoning. We found no correlation with total positive psychotic symptoms [rho(24) = −0.08, *P* = 0.69, including the “P2: conceptual disorganization” item, rho(24) = −0.07, *P* = 0.73], nor with total depressive symptoms [rho(24) = −0.15, *P* = 0.46, Spearman’s correlation]. There was no monotonic relationship between Δω and other measured cognitive variables in PScz [i.e., working memory capacity on digit span task: rho(24) = 0.18, *P* = 0.38; estimated full scale IQ: rho(24) = 0.12, *P* = 0.56, Spearman’s correlation].

### Relationship to Neurophysiological Signatures of Structured Memory Replay.

**Fig. 6.**
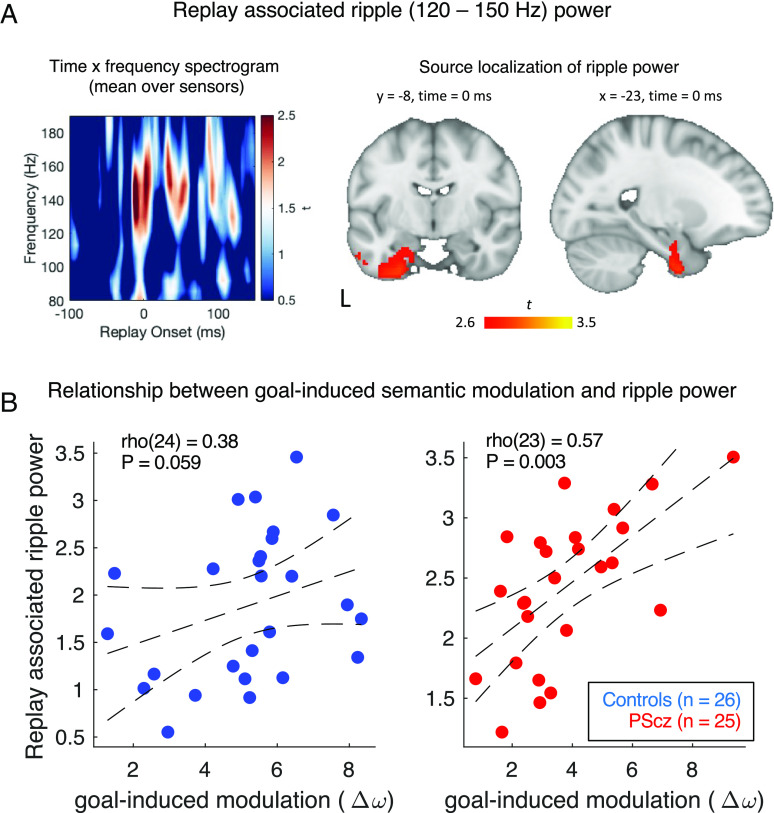
Relationship to replay associated ripple power. (*A*) Ripple power at replay onset. *Left:* In our previous MEG study in the same sample, we detected a transient increase of high frequency (ripple) power at time points displaying high evidence for spontaneous neural replay of a previously learned task structure, in MEG data from a post-task rest session (see *SI Appendix* and ref. 11). Spectrogram shows mean power change following onset of putative replay events (0 ms, see *SI Appendix*) (mean over all replay events and sensors), where power is normalized to a pre-onset baseline (−100 to −10 ms) for each frequency band (as in ref. 11). T-values at each time × frequency combination derived from two-tailed one-sample *t* test over all participants (n = 51). *Right:* Source localization of ripple power increases at time of putative replay onset (0 ms in spectrogram) using a beamforming analysis limited to 120 to 150 Hz power band. Ripple power at replay onset was source localized to HEC (significant cluster at P_FWE_ < 0.05, whole-brain level, cluster-based permutation test, 5,000 permutations). Figure shows the statistical parametric map for a group level analysis, displaying the voxel-wise evidence (t-value) that ripple power at replay onset (0 ms) originates from each intracranial source (see *SI Appendix* for further details). (*B*) Across participants replay-associated ripple power relates to a behavioral signature of goal-induced semantic modulation ( Δω ). [Replay-associated ripple power defined as peak power increase within the ripple-band from 0 to 50 ms (±10 ms) immediately following replay onset, as in ref. 11]. Spearman’s correlation, two-tailed. Trend lines depict 95% CI of line of best fit. This relationship is also significant in the combined sample of PScz and controls (*Results*). All results restricted to n = 51 participants who completed both verbal fluency and MEG.

The functions of HEC include a strong link to associative cognition (15, 17, 18, 20, 50–53). This motivated us to test the relationship between goal-induced semantic modulation ( Δω   ) and a neural index of associative memory reactivation, thought to originate from HEC. Here, we used resting-state MEG data collected from the same sample of participants from an experimental session conducted on another day (typically, the day after verbal fluency). The full MEG session involved a sequence learning task, wherein participants were tasked to learn the sequential relationships between eight task pictures and maintain this knowledge during a 5-min post-task resting-state scan (11). Using multivariate neural decoding, we previously showed that in this post-task resting MEG data, whole-brain activity patterns reflect spontaneous neural replay of learned task sequences (from the preceding task), where putative “replay onsets” are coincident with transient increases in high-frequency power (ripple band, 120 to 150 Hz) emanating from HEC sources (Fig. 6*A*) (11, 18). Preclinical studies show that these replay-associated ripple events are causally implicated in associative learning and cognitive map stabilization (54–58).

We correlated behavioral measures of semantic clustering during verbal fluency (i.e., Δω ) and MEG-derived measures of replay and ripples across participants, reasoning that the latter might index a trait-level capacity for associatively-structured memory reactivations. Across all participants, we found a positive relationship between goal-induced semantic modulation ( Δω ) and MEG-derived measure of replay-associated ripple power, with no group difference in the slope of this relationship [ripple ~ group * Δω multiple regression: $\beta_{\Delta \omega }$ = 0.16 ± 0.047, t(47) = 3.39, *P* = 0.001. $\beta_{\mathrm{group}}$ = −0.48 ± 0.47, t(47) = −1.03, *P* = 0.31. $\beta_{\mathrm{group}*\Delta \omega }$ = −0.06 ± 0.09, t(47) = −0.67, *P* = 0.51]. (Here, “replay associated ripple power” is defined as the peak power increase in the range 120–150 Hz within 50 ms of a replay onset [±10 ms], averaged over all MEG sensors, as in ref. 11.) Thus, participants exhibiting the greatest ripple power at the time of putative replay showed a greater tendency to use semantic information to guide word selection in a context-sensitive manner. The correlation between ripple power and Δω was also significant in PScz alone [rho(23) = 0.57, *P* = 0.003, Spearman’s correlation, Fig. 6*B*]. We note that this same MEG signature of ripple power was previously shown to correlate with the strength of a neural representation of learned task structure (11).

To probe this brain–behavior relationship further, we tested the association between replay-associated ripple power and model-agnostic behavioral measures (i.e., semantic and orthographic path optimality measures from both tasks). Replay-associated ripple power was linearly related to semantic path optimality in the category fluency task [global optimality divergence: $\beta_{\mathrm{optimality\_divergence}}$ = −0.24 ± 0.062, t(47) = −3.87, *P* = 0.0003; local optimality divergence: $\beta_{\mathrm{optimality\_divergence}}$ = −0.30 ± 0.087, t(47) = −3.44, *P* = 0.001; results from ripple ~ group * optimality_divergence multiple regression across all participants]. However, there was no significant relationship between ripple power and semantic path optimality in the letter task, nor in either task when considering path optimality defined using an orthographic distance metric. This indicates that a primary contributor of the brain-behavior correlation seen with Δω stems from variance in semantically constrained sequencing.

We found no correlation between Δω and our previously reported measure of spontaneous replay, where the latter quantifies the degree to which resting-state MEG data manifest a temporal ordering of neural state reactivations that recapitulates an inferred task structure at 40 to 50 ms lag (11) [replay ~ group * Δω multiple regression: $\beta_{\Delta \omega }$ = 0.05 ± 0.14, t(47) = 0.38, *P* = 0.71. $\beta_{\mathrm{group}}$ = 3.09 ± 1.37, t(47) = 2.26, *P* = 0.03. $\beta_{\mathrm{group}*\Delta \omega }$ = −0.54 ± 0.29, t(47) = −1.95, *P* = 0.06].

In a control analysis, we found no significant relationship between Δω and replay-associated alpha power [defined identically to replay-associated ripple power, but using the alpha frequency band, 8 to 13 Hz] [alpha ~ group * Δω multiple regression: $\beta_{\Delta \omega }$ = −0.051 ± 0.029, t(47) = −1.74, *P* = 0.09. $\beta_{\mathrm{group}}$ = 0.041 ± 0.288, t(47) = 0.143, *P* = 0.89. $\beta_{\mathrm{group}*\Delta \omega }$ = 0.031 ± 0.059, t(47) = 0.52, *P* = 0.61]. Using a similar multiple regression approach, we also found no significant main effect of Δω on dynamical properties of ripples during MEG rest data, where the latter includes mean (and SD) duration of ripple events (defined as epochs exhibiting high ripple power) and mean (and SD) time separating ripple events (see *SI Appendix* for details).

### Relationship to Medication Status.

PScz taking and not taking dopamine 2/3 receptor antagonist (antipsychotic) medication did not differ in Δω [PScz on medication: 3.57 ± 0.60, off medication: 3.70 ± 0.55, z(24) = −0.51, *P* = 0.61, Wilcoxon rank sum test, two-tailed], total negative symptom severity [total PANSS negative symptom score, PScz on medication: 14.5 ± 1.64, off medication: 12.7 ± 1.92, z(24) = −1.11, *P* = 0.27, Wilcoxon rank sum test, two-tailed], mean number of response items (expressed as context-specific rank) [PScz on medication: 25.2 ± 2.94, off medication: 24.3 ± 3.64, t(24) = 0.201, *P* = 0.84, two sample *t* test, two-tailed], nor replay-associated ripple power [PScz on medication: 2.39 ± 0.18, off medication: 2.47 ± 0.19, t(24) = −0.304, *P* = 0.76, two sample *t* test, two-tailed].

## Discussion

At the behavioral level, we present convergent evidence that schizophrenia is associated with reduced semantically guided memory sampling during a category fluency task, even after controlling for between-participant differences in list length or contents (e.g., vocabulary). Using computational modeling, we show that the relative weighting of semantic and orthographic information on task performance is highly sensitive to task goal (context), and that the degree of this “goal-induced semantic modulation” ( Δω ) is reduced in schizophrenia. Strikingly, between-participant variance in Δω correlated with an expression of symptom severity that relates to abstract conceptual reasoning and a “negative” dimension of formal thought disorder (5).

Our work avails of novel NLP tools for automated analysis of speech. Such tools have previously been applied to speech samples from patients with psychosis to derive computational metrics with diagnostic and prognostic utility (9, 28–31, 59–66). For example, measures of semantic coherence (e.g., cosine distances between consecutive words, embedded in semantic space) can potentially be used to differentiate PScz and healthy controls (30, 43, 67–70), automate identification of formal thought disorder in PScz (43, 67, 71, 72), and predict transition to psychosis in those experiencing prodromal symptoms (59, 61). Notwithstanding these proof-of-concept findings, single studies tend to test a variety of hypotheses, and the pattern of statistically significant findings is often inconsistent between studies. Studies also differ in approaches for operationalizing semantic coherence, elicitation of speech data, and clinical assessment of thought disorder, impeding knowledge synthesis (31, 73).

Our study departs from the aforementioned research in that we test a neurocognitive hypothesis where we propose that differences in verbal fluency relate to cognitive and neural processes that underpin associative (relational) cognition. Thus, in addition to our behavioral findings, we find a relationship between Δω   and an MEG measure of replay-associated ripple power, where the latter is measured during a rest session following a separate sequence learning task. We consider this intriguing given an established role for both HEC and hippocampal ripples in associative memory and reasoning across diverse task domains (e.g., spatial memory, non-spatial structure learning, semantic retrieval) (10, 14–16, 18, 19, 50, 53, 74, 75). Although our cognitive and neural measures were derived from different task paradigms (semantic memory search and non-spatial sequence learning, respectively), under an HEC “cognitive map” hypothesis they are thought to engage similar neural circuits that support reasoning about the relationships between entities (14, 15). However, we acknowledge that any claim of functional significance or cognitive specificity needs to be tempered, given the correlational and indirect nature of the findings.

We did not find any monotonic relationship between Δω and neural replay across participants, where replay might be considered a more specific measure of sequential neural reactivation (compared to ripples). Of note, both replay and ripples were measured during a rest period with reference to a separate inference task, typically acquired on a different day to verbal fluency data. Although we caution against overinterpretation of the null replay result, one speculation is that expression of replay within an individual might be particularly sensitive to behavioral context (i.e., behavioral “state”), whereas a propensity to generate ripples might reflect a more “trait-like” participant property (related to HEC circuit organization) that underpins computations common to divergent tasks. In our context, the differing cognitive demands of verbal fluency and sequence learning tasks might engender qualitatively different sequence generation (i.e., replay) regimes in HEC (53) (“diffusive” in verbal fluency, vs. fixed sequential in sequence learning), such that mechanisms of sequence generation underlying performance of one task are not readily transferrable to those underlying another. Future experiments involving measurement of ripples and replay across multiple tasks in the same participants are required to test this hypothesis.

We found a correlation between “goal-induced semantic modulation” ( Δω   ) and negative symptoms. Some studies have reported a significant correlation between related semantic coherence measures and positive symptoms, including formal thought disorder (43, 67, 71, 72). However, such findings are not universally reported, and, as noted above, previous studies have used a variety of task contexts to elicit verbal data, complicating interpretation given the task-dependent effects on NLP speech metrics reported both in the present work and elsewhere (30, 66). Moreover, in contrast to the present work, many previous studies that use category fluency tasks (43, 67, 69, 71, 72) adopt short task durations (mean list length <20 words), have not controlled for between-subject differences in recalled items, and do not address questions of specificity for semantic associations and sensitivity to task context.

Limitations of the present work include the fact that we assume that the pre-trained word embedding model, fixed for all participants, approximates internal semantic representations in PScz and control participants to a similar degree. Here, we note that our measure of semantic similarity is equivalently able to predict inter-item retrieval times in PScz and control samples (Fig. 4*B* and *SI Appendix*). Second, our computational model, while accounting for participant differences in vocabulary, does not address potential influences of non-specific cognitive processes such as working memory or self-monitoring. Importantly, however, we find no correlation between cognitive trait variables and fitted model parameters. Our model postulates that word selection unfolds as a “local search” in semantic space. Other hypotheses include a random walk on a semantic graph (76) or a two-stage process alternating between a local search and “patch switch” policy (21, 22, 43, 69). The latter two-stage modeling approach necessitates further analytic decisions regarding how patch switches are defined, complicating interpretation. Notwithstanding this concern, in *SI Appendix*, we show that our behavioral findings remain significant after amending our model to mimic such a two-stage process. More generic limitations include the correlational nature of our analyses, the fact that behavioral and neural measures were acquired at different times, and a relatively small sample.

In summary, our findings offer tentative support for an hypothesis that some symptoms of schizophrenia may reflect a dysregulation of a neurocognitive process involving structured conceptual representations (cognitive maps). We anticipate that future studies, combining concurrent functional neuroimaging, language tasks, and rapidly advancing NLP word-embedding tools, will continue to shed light on the neural coding schemes that support such associative cognition.

## Materials and Methods

### Participants and Assessment.

The study was approved by the London Westminster NHS Research Ethics Committee (15/LO/1361). All participants provided written informed consent and were compensated for their time. Participants attended for two visits (verbal fluency behavioral session, and MEG task session, approximately 1 d apart) (11). The final sample for the verbal fluency analysis comprised 52 participants [26 PScz (6 female, range 18 to 45 y, 13 not taking any psychiatric medication) and 26 control participants (6 female, range 18 to 45 y), see *SI Appendix*, Table S1]. One patient participant declined MEG owing to paranoia. See *SI Appendix* for full details.

### Verbal Fluency Task and Analysis.

On visit 1, participants completed two verbal fluency tasks (order randomized), wherein they were asked to name as many words as they could either belonging to the category “animals” (category fluency) or starting with the letter “p” (letter fluency) in 5 min. We used a fastText (Facebook AI Research) pretrained NLP word embedding model (40) to quantify the semantic association (proximity) between each pair of words in terms of cosine similarity/distance (Fig. 1*A*). We defined an analogous (non-semantic) measure of orthographic (letter-by-letter) association as the Levenshtein distance (48).

For both conditions, we quantified the degree to which the sequence of emitted words (the “trajectory through semantic space”) approximated the optimal sequence that minimizes total traversed semantic (or orthographic) distance [i.e., a modified Travelling Salesman optimization problem (77), see *SI Appendix*] (Fig. 3*A*). We defined “global optimality divergence” as the difference between the observed and optimal path total distance, and “local optimality divergence” as the degree to which optimal and observed trajectories are aligned at the level of individual item-item transitions. Crucially, we express both effects as a z-score with respect to a participant-specific permutation-derived null distribution, thus controlling for participant differences in vocabulary and list length [i.e., z-scores of 0 indicate a random word search selection process, which is insensitive to semantic (or orthographic) associations; negative z-scores reflect word selection processes that are predictable given such associations]. See *SI Appendix* for further discussion and mathematical details.

To gain insight into processes driving task performance, for each participant, we fitted a family of generative computational models to the concatenated word list data from both tasks using maximum likelihood estimation (as in ref. 21). We identified the winning model using Akaike Information Criteria (AIC) at the group level (78). See *SI Appendix* for mathematical details, model comparison, and parameter recovery.

### MEG Task and Analysis.

On visit 2, participants completed a separate sequence learning task during MEG, as described in our previous report (11). Briefly, during MEG participants learned sequences containing eight task pictures, before completing a 5-min resting-state MEG scan. We previously identified time points in MEG data form this post-task rest scan that exhibited high evidence of sequential neural replay (i.e., spontaneous neural reactivations of task state representations that recapitulated the task transition structure, occurring at 40 ms lag) using temporally delayed linear modeling (TDLM) (79). Such time points were associated with an exuberance of high frequency (120 to 150 Hz, “ripple”) power, identified as originating from HEC sources in a beamforming analysis (11). In the present work we extract, for each participant, a single estimate of replay strength (evidence of replay at 40 to 50 ms replay time lag) and replay-associated ripple power [peak power in 120 to 150 Hz band, in the 0 to 50 ms (±10 ms) window following putative replay events], for all brain–behavior correlations. These MEG measures are identical to those reported in our previous MEG replay study (11). See *SI Appendix* for full details of MEG task and analysis.

### Statistical Analysis.

We used parametric and non-parametric statistical tests for normally and non-normally distributed variables, respectively (normality was tested using a Shapiro–Wilk test). Effects are reported as mean ± 1 standard error of mean (SEM), and two-tailed *P* < 0.05 is deemed significant throughout. See *SI Appendix* for further details.

## Supplementary Material

Appendix 01 (PDF)

## Data Availability

Analysis code and data to reproduce the results in the paper will be made available at github.com/matthewnour/verbal_fluency_trajectories and github.com/YunzheLiu/TDLM. The manuscript additionally relates behavioral measures to MEG data originally presented in a previous study: (11).
