# Supplementary material for: Trajectories through semantic spaces in schizophrenia and the relationship to ripple bursts
Source: Proc Natl Acad Sci U S A. 2023 Oct 10;120(42):e2305290120. doi: 10.1073/pnas.2305290120 (PMC10589662; doi:10.1073/pnas.2305290120)
Supplement: Supplementary file 1 — Appendix 01 (PDF) [file pnas.2305290120.sapp.pdf]

PNAS

Supporting Information for

Trajectories through semantic spaces in schizophrenia and the relationship to ripple bursts

Matthew M Nour[1,2]*, Daniel C McNamee[3], Yunzhe Liu[4,5], Raymond J Dolan[2,4,6]

[1.] Department of Psychiatry, University of Oxford, Oxford, OX3 7JX, UK

[2.] Max Planck University College London Centre for Computational Psychiatry and Ageing Research, London, WC1B 5EH, UK

[3.] Champalimaud Research, Centre for the Unknown, 1400-038 Lisbon, Portugal.

[4.] State Key Laboratory of Cognitive Neuroscience and Learning, IDG/McGovern Institute for Brain Research, Beijing Normal University, Beijing, 100875, China

[5.] Chinese Institute for Brain Research, Beijing, 102206, China
[6.] Wellcome Centre for Human Neuroimaging, University College London, London, WC1N 3AR, UK

* **Correspondence:** Matthew M Nour, matthew.nour@psych.ox.ac.uk

This PDF file includes:

Supporting text
Figures S1 to S5
Table S1
SI References

Supplemental Methods

Participants and assessment

The study was approved by the London Westminster NHS Research Ethics Committee (15/LO/1361). All participants provided written informed consent and were compensated for their time. We initially recruited a total of 31 PScz (6 female, 25 male) with schizophrenia (assessed with the Structured Clinical Interview for DSM-IV-TR, Axis I Disorders, SCID-I (1)) from London community psychosis NHS clinics, and 29 healthy volunteers (6 female, 23 male), from the same geographical area through online advertisements. Groups were matched for age, gender, IQ and educational attainment. General exclusion criteria were anticonvulsant or benzodiazepine medication, age > 45 years, poor vision limiting performance and not having been educated in English. Healthy volunteers were not taking neurological or psychiatric medication, had no history of neurological or psychiatric disorder (assessed by SCID-I (1)), and no family history of psychosis. PScz had no neurological or psychiatric comorbidity. Participants attended for two study visits. Visit 1 comprised verbal fluency tasks, cognitive and clinical assessments, and MEG pre-training. Visit 2 (typically 1 day later) comprised MEG task (as described in (2)).

Of the recruited participants, 53 completed verbal fluency tasks on visit 1 (26 PScz [6 female, mean age 28.5 years, range 21 – 40 years, 13 not taking D2/3 antagonist medication, 2 medication naïve] and 27 controls [6 female, mean age 27.7 years, range 18 – 45 years), of whom 52 additionally completed the MEG task on visit 2 (1 PScz [medication naïve] declined due to paranoia). We identified outliers as participants who generated word lists containing a number of repetitions that exceeded the group median by >3.5 standard deviations, and excluded them from all analyses (1 control participant, who met outlier criteria for both ‘category’ [10 repetitions] and ‘letter’ [18 repetitions] tasks). See **Table S1** for final sample demographic and clinical / cognitive scores.

We assessed psychiatric symptoms with the Positive and Negative Syndrome Scale (PANSS) scale (3), Montgomery Åsberg Depression Rating Scale (MADRS) (4), and General Assessment of Function (GAF) (5). We administered brief measures of IQ (the

Wechsler Test of Adult Reading, WTAR (6)) and working memory (mean of forward and backward Digit Span).

Pre-processing of verbal fluency data

Pre-processing was identical for 'category' and 'letter' tasks. Item lists for each participant were generated from transcribed verbal responses. We first removed all items that did not have a semantic embedding (i.e., non-words, e.g., 'pozzy'. 'Category' task: no responses met this criterion. 'Letter' task: control: mean = 0.23 non-words per list ± 0.08 SEM, PScz: mean = 0.38 ± 0.19, $z(50) = 0.17$, $P = 0.87$, Wilcoxon rank sum test, two tailed), and all words that were deemed invalid under the task rules (i.e., non-animals for 'category' task: control: mean = 0.00 ± 0.00, PScz: mean = 0.12 ± 0.06, $z(50) = -1.74$, $P = 0.08$, Wilcoxon rank sum test, two tailed, and non-'p' words for 'letter' task controls: mean = 0.08 ± 0.05, PScz: mean = 0.08 ± 0.05, $z(50) = 0.00$, $P = 1.00$, Wilcoxon rank sum test, two tailed). We removed consecutive word perseverations for all analyses. Non-consecutive word repetitions were additionally removed for optimal path analyses (as identified optimal paths, by definition, cannot include re-visits to the same response item).

We used a semi-automated approach to extract inter-response retrieval times (RT) corresponding to each response item for each participant (used for supplementary analyses). We first computed the amplitude envelope of the audio time series data corresponding to each participant's verbal responses (after down-sampling this time series to 100 Hz and smoothing with a 50 ms Gaussian window). We then used an automated peak detection algorithm to identify time points of transiently high amplitude (i.e., candidate start times of a new response item) (https://terpconnect.umd.edu/~toh/spectrum/PeakFindingandMeasurement.htm). Candidate start times were then inspected manually one-by-one to identify the events that corresponded to the beginning of the first syllable of each transcribed response item.

Word embedding and semantic distance quantification

To quantify semantic association each response item was first represented as a vector in a common multidimensional semantic space (as in (7–10)) (**Figure 1A**). We used a fastText word embedding model (Facebook AI Research), which had been pre-trained on a 16 billion token corpus of English language text (internet corpus, including Wikipedia

2017, UMBC webbase corpus and stat.org news dataset), as implemented in the MATLAB Text Analytics toolbox (*fastTextWordEmbedding*) and available from https://fasttext.cc/docs/en/english-vectors.html (*wiki-news-300d-1M.vec.zip*). This model contains a 300 dimension vector representation for ~1 million English words (11).

Briefly, training predictive word embedding models starts form an assumption that the semantic association between a pair of words is reflected in their co-occurrence statistics in natural language (i.e., the degree to which they are found together in sentences, documents, or text windows of a fixed number of words). A general approach for a 'continuous bag of words' model is to train a simple neural network, which takes a natural language 'context' as input (e.g., the collection of words that are contained in a fixed size window centered around the target word, window size 5 – 15 (11)) and outputs a prediction for the target word. Words in the input and output layer may be represented as one-hot vectors (or multi-hot vectors for multi-word context inputs), such that the dimensionality these inputs and outputs is equal to the number of unique words (tokens) in the training set. A fully-connected hidden layer, however, has a much reduced dimensionality (e.g., 300 units), imposing an information bottleneck. On completion of training, a word's 'embedded' representation is equal to the [300, 1] dense vector of connection weights from the 'hot' input unit, to each node of the hidden layer (12). This approach thus embeds the high dimensional input representation within a much lower dimensional space that is optimal for predicting word co-occurrence in natural language. Stemming from this objective, distances between word vectors in the learned low dimensional representation capture a semantic association between words (13, 14).

The cosine of the angle between two word embedding vectors (i and j) is a proxy for the semantic similarity between words ($s_{i,j}^{sem}$, scaled between 0 and 1):

$$s_{i,j}^{sem} = cos\theta_{i,j} = \frac{\langle i,j \rangle}{\|i\|\|j\|} \tag{1}$$

, where $cos\theta_{i,j}$ is the normalized dot product of vectors i and j. $s_{i,j}^{sem}$ can be used to populate an item-item similarity weight matrix, $\mathbf{S}^{semantic}$, for each participant (**Figure 1B**), and

permits the analyses of item lists as trajectories in a common semantic space (**Figure 1C**). Semantic distance is defined as:

$$d_{i,j}^{sem} = 1 - s_{i,j}^{sem} \qquad (2)$$

We also defined a measure of orthographic distance between item pairs, $d_{i,j}^{orth}$, using the Levenshtein distance between words (15) (this quantifies the number of single character edits to transform one character string into another, where permitted edits are character insertions, deletions, and substitutions). $d_{i,j}^{orth}$ was scaled between 0 and 1 (using the distribution of distances between all item pairs). We defined orthographic similarity as

$$s_{i,j}^{orth} = 1 - d_{i,j}^{orth} \qquad (3)$$

We operationalized distances involving multi-word response items (e.g., distance between 'killer whale' & 'shark') as the mean of the distances between the constituent words of both items (e.g., *S('killer', 'shark')* & *S('whale', 'shark')*).

Importantly, the correlation between semantic and orthographic distance was low ('category' task: r(138754) = 0.04, P < 0.001, 'letter' task: r(953559) = 0.11, P < 0.001, Pearson's correlation across all word pairs).

Trajectory effect size quantification

We used a within-participant permutation approach to quantify the degree to which the sequential order of generated word lists reflected distances between adjacent words (vs. a random order), in a manner that controlled for between-participant differences in composition and length of word lists (using within-participant z-scoring and a sliding-window approach, respectively). Specifically, for each participant we calculated the degree to which measures of sequential sampling deviated from those expected under a null distribution, generated by a random sampling policy on the set of words emitted by the participant (i.e., the z-score of observed effect vs. list-specific empirical null distribution generated from 1000 permutations, as in **Figure S1** (10, 16)). Notably, participants also differed in the total number of words generated within 5 minutes, and list length may confound our z-score effect size measure. To address this potential confound, whenever

reporting z-score effects (i.e., optimality divergence and community trajectories) we use a sliding window approach that allows us to estimate effects of interest using word lists of identical length for each participant, while still making use of the complete data from each participant (window size = minimum list length across participants, stride length = 1). Thus, we calculate the (z-scored) effect of interest for each window, and then calculated the mean of this effect over all windows (analogous to (16, 17)).

Optimal trajectory identification

To identify optimal search paths for each participant we implemented the Travelling Salesman optimization algorithm using linear integer programming in MATLAB (https://uk.mathworks.com/help/optim/ug/travelling-salesman-problem.html). Here, for an observed sequence of n words, S, containing no duplicates, our goal was to identify the path that starts with s_1 (the 1st word in the observed list), and visits each other word in S exactly once, with the lowest sum of edge weights (undirected edge weights between words denote distances in a given metric: semantic or orthographic). The standard Travelling Salesman problem concerns the identification of shortest *cycles* (i.e., paths that start and end with s_1, visiting each other word only once), and as such is not perfectly aligned with our objective. However, by including an additional 'dummy' word s_{dummy}, which has a very small non-zero directed edge weight to s_1 (the 1st observed word in the list) and from s_n (any other chosen word in the list), and no permissible edges to any other word in S, we force the algorithm to identify an optimal (shortest) path that starts at s_1 and ends with s_n (i.e., a cycle containing $s_n \rightarrow s_{dummy} \rightarrow s_1$). For a list of length n we implemented this modified optimization algorithm $n - 1$ times, on each occasion assigning the end state (s_n) as a different (non-s_1) word (s_1 is fixed as the true observed 1st word in the list). We define the 'optimal path' as the shortest path of this set (i.e., lowest sum of distances).

We then define the 'global optimality divergence' as the difference between the total (semantic) distance traversed in the observed word sequence vs. that identified by the modified Travelling Salesman procedure (i.e., the 'optimal' sequence). We also define a measure of 'local optimality divergence' for each analysis window. Here, for each adjacent

item pair in an observed list we identified the number of edges separating items in the optimal trajectory (i.e., the geodesic distance), and calculated the sum of this divergence metric across all transitions in the observed list.

As outlined above, for each participant list we express effects (optimality divergences) as z-scores of the observed value with respect to participant-specific permuted lists, and nest the entire analysis within a sliding window outer loop, ensuring that effects from all participants are derived using list length windows of the same size.

Computational modelling of behavior

In computational modelling we considered a family of generative models that construed the sequential selection of words as a local search process in internal memory space, in which the association between items (i.e., the topology of the space) is defined by orthographic and/or semantic similarity, and where the probability of selecting a word is a monotonic function of its proximity to the previously emitted item (18, 19). Specifically, the probability of observing the *t^th^* emitted word (W_t) is derived from a softmax function of the association strength between W_t and the previous emitted word (i.e., $S(W_{t-j}, W_t)$), where the softmax is normalized by the set of all unique N words emitted by over all participants. This similarity function (semantic or orthographic space) is fixed across participants, but scaled by a multiplicative salience parameter (β) which varies across participants (i.e., a free parameter) (18, 19):

$$P(W_t|W_{t-1}) = \frac{e^{\beta\, S(W_{t-1},W_t)}}{\sum_{k=1}^{N} e^{\beta\, S(W_{t-1},W_k)}} \tag{4}$$

We fitted 5 such models, instantiating different hypotheses regarding the relative influence of semantic and orthographic association in task performance, and how this varies between 'category' and 'letter' fluency tasks.

The simplest 3 models contain separate β parameters for word lists relating to 'category' and 'letter' tasks (i.e., 2 free parameters), but mandate that in each task the softmax response function depends on semantic ($S_{sem}(W_{t-1}, W_t)$) or orthographic

($S_{orth}(W_{t-1}, W_t)$) similarity alone. These models are therefore equivalent to fitting a separate softmax model to ‘category’ and ‘letter’ fluency data separately.

The first (‘semantic’) model uses the semantic similarity for both ‘category’ and ‘letter’ tasks:

$$P(W_t|W_{t-1}) = \frac{e^{\beta_{sem}\, S_{sem}(W_{t-1},W_t)}}{\sum_{k=1}^{N} e^{\beta_{sem}\, S_{sem}(W_{t-1},W_k)}} \tag{5}$$

A second (‘orthographic’) model uses orthographic similarity for both tasks:

$$P(W_t|W_{t-1}) = \frac{e^{\beta_{orth}\, S_{orth}(W_{t-1},W_t)}}{\sum_{k=1}^{N} e^{\beta_{orth}\, S_{orth}(W_{t-1},W_k)}} \tag{6}$$

Finally, a third (‘task congruent’) model used semantic similarity for the ‘category’ task data, and orthographic similarity for ‘letter’ task data, guided by our model-agnostic findings.

$$P(W_t|W_{t-1}) = \frac{e^{\beta_{task}\, S_{task}(W_{t-1},W_t)}}{\sum_{k=1}^{N} e^{\beta_{task}\, S_{task}(W_{t-1},W_k)}} \tag{7}$$

(‘task’ is assigned to ‘semantic’ for the ‘category’ fluency task data and ‘orthographic’ for the ‘letter’ fluency task data, similar to (9)).

Importantly, these 2-parameter models do not permit any arbitration between semantic and orthographic similarity in task performance. To capture such an arbitration, we defined 2 additional models in which the selection of the tth word is a function of both semantic and orthographic similarity, and where the relative weighting of these association strengths can vary between ‘category’ and ‘letter’ tasks. The first of these arbitration models contained a single salience parameter that was held constant over both tasks, and a

weighting parameter governing the relative contribution of semantic and orthographic similarity, fitted separately for 'category' and 'letter' tasks (i.e., 3 free parameters).

$$P(W_t|W_{t-1}) = \frac{e^{\beta_{gen}\, S(W_{t-1},W_t)}}{\sum_{k=1}^{N} e^{\beta_{gen}\, S(W_{t-1},W_k)}} \quad (8)$$

where,

$$S(W_{t-1}, W_t) = \beta_{weight}\, S_{sem}\left(W_{t-1,} W_t\right) + \left(1 - \beta_{weight}\right) S_{orth}\left(W_{t-1,} W_t\right) \quad (9)$$

The second arbitration model fitted separate semantic and orthographic salience parameters for 'category' and 'letter' fluency tasks (i.e., 4 free parameters).

$$P(W_t|W_{t-1}) = \frac{e^{\,\beta_{sem}\, S_{sem}(W_{t-1},W_t)+\beta_{orth}\, S_{orth}(W_{t-1},W_t)}}{\sum_{k=1}^{N} e^{\,\beta_{sem}\, S_{sem}(W_{t-1},W_k)+\beta_{orth}\, S_{orth}(W_{t-1},W_k)}} \quad (10)$$

On each response item we first mapped similarity measures $S(W_{t-1}, W_k)$ to a uniform distribution on the interval (0, 1] prior to calculation of $P(W_t|W_{t-1})$ (using the empirical cumulative probability distribution of $S(W_{t-1}, W_k)$ measures over all words k; akin to conducting model fitting using the rank-ordered association between the previous word and all candidate words).

We fitted all models to participant lists (concatenated over tasks) using maximum likelihood estimation, and for each participant quantified the Akaike information criterion (AIC) for each model,

$$AIC = 2k - 2\ln(\hat{\mathcal{L}}) \quad (11)$$

where k indicates the number of estimated free parameters in the model (penalizing for model complexity), and $\hat{\mathcal{L}}$ is the maximum of the likelihood function for the model. We identified the winning model at the group level as that yielding the lowest summed AIC

over participants (of note, the 4P model described in eq. (10) is also the winning model in PScz and control samples separately).

To assess the parameter recoverability of the winning arbitration model (4P) model (eq. (10)), we simulated word list data from 100 experiments, in each generating word lists (length = 50) for 25 'participants', where each participant exhibits a unique combination of β_{sem} and β_{orth} values (corresponding to different red asterisk positions in **Figure S4A**). We then fitted the winning arbitration model to each simulated participant's word list and compared the recovered parameters to the ground truth (generative) parameters. Note that the winning arbitration model (4P model, eq. (10)) in effect can be considered as two separate 2P arbitration models, applied to each task independently (unlike the 3P model, which retains a common general salience parameter spanning both tasks, eq. (8)).

The models above conceive of semantic search as a 'local' search process, sensitive only to association strength between a candidate response (t), and the previous response (t-1). Previous analyses have considered that such local search governs task performance only when participants are engaged in selecting items from within a 'semantic community' (e.g., 'fish', 'shark', 'whale'), while inter-community 'switch' responses (e.g., 'whale', 'ant') are instead governed by more global (non-semantic) salience cues, such as word frequency (8, 9, 19). To test whether our model-derived results were robust to exclusion of 'inter-community' responses, we re-fitted our winning computational model (eq. (10)) , censoring the contribution of an observed word (t) to the likelihood estimation if the community assignment of (t) was not identical to that of item (t-1) (see **Identifying community structure in animal space**, and **Supplemental Results**).

Identifying community structure in animal space

We used the Louvain agglomerative clustering algorithm (20) in combination with a consensus clustering procedure (21) to partition the semantic similarity matrix of 'category' task words (all unique words over participants) into the set of non-overlapping communities, c. The algorithm seeks a partition that maximizes the modularity statistic (Q), which is a measure of network segregation that quantifies the difference between the density of within-community connection weights and the density that would be expected

under a null model that preserves the distribution of node strengths in the observed network (20, 22, 23). For positive undirected weighted networks, it is defined as:

$$Q = \frac{1}{2v}\sum_{ij}\left(s_{ij} - \lambda\frac{k_i k_j}{2v}\right)\delta(c_i, c_j) \tag{12}$$

, where i and j refer to individual network nodes (words), v is the total sum of unique connection weights (semantic similarities) between all nodes in the network ($v = \frac{1}{2}\sum_{ij} s_{ij}$), s_{ij} is the observed connection weight between nodes i and j, $\frac{k_i k_j}{2v}$ is the connection weight between i and j that would be expected under a null model respecting node-specific strength ($k_i = \sum_j s_{ij}$), and $\delta(c_i, c_j)$ is an indicator (Kronecker delta) function set to 1 when c_i=c_j (i.e., when word i and j are assigned to the same community), and 0 otherwise (20, 22). The resolution parameter, λ, tunes the threshold for community detection by scaling the influence of the null model, such that larger values result in partitions with an increased number of smaller communities (23). We set λ as 1, and present supplementary results showing that our results are relatively robust to a change in this value (**Figure S5**). The Louvain algorithm was implemented in the MATLAB Brain Connectivity Toolbox (https://www.nitrc.org/projects/bct/) version 2017-15-01.

Given the stochastic nature of the Louvain algorithm we used a consensus clustering approach to ensure the robustness of the final community structure (21, 23). Specifically, we iteratively applied the algorithm 1000 times with different initial random seeds. This generated 1000 separate partitions, which were then combined to a single agreement matrix, *D*, where entry d_{ij} represents the proportion of partitions in which nodes (words) i and j were assigned to the same community. Following a thresholding step (in which all entries <50% agreement were set to zero) (24, 25), the agreement matrix was then subjected to another 1000 iterations of the Louvain algorithm. This procedure was repeated until each of the resulting 1000 partitions was equal (i.e., a consensus partition). Prior to running community detection, we set $s_{ij} = 0$ for all $i = j$, and ensured that the network was symmetrical (input matrix = $\frac{1}{2}\mathbf{SS}^{\mathrm{T}}$) such that $s_{ij} = s_{ji}$.

MEG sequence learning task and analysis

The MEG task and analysis details are outlined in detail in Nour et al,. (2021) (2), and are described here only briefly.

During MEG (visit 2), participants performed a non-spatial sequence learning ('Applied Learning') task, previously shown to elicit neural replay during a post-task rest session (2, 26). During the Applied Learning task sessions participants needed to infer the sequential relationships between 8 task pictures ([A→B→C→D] & [A'→B'→C'→D']). MEG contained two additional sessions of relevance. First, a Stimulus Localizer task prior to Applied Learning, wherein we presented each task picture in a random order (1 s presentation, ~ 40 - 52 presentations per picture), to obtain visually-evoked MEG data for training stimulus decoders. Second, a 5 minute eyes-open rest session immediately after Applied Learning, wherein we quantified spontaneous neural replay of inferred task sequences.

MEG was recorded continuously at 1200 samples/second using a whole-head 275-channel axial gradiometer system (CTF Omega, VSM MedTech), while participants sat upright (3 sensors not recorded due to excessive noise in routine testing). Sensor data were high-pass filtered at 0.5 Hz using a first-order IIR filter, and downsampled to 100 Hz (sequenceness analysis) and 400 Hz (time-frequency analysis). Excessively noisy segments and sensors automatically identified and removed from the data. Independent Component Analysis (FastICA, http://research.ics.aalto.fi/ica/fastica) was used to decompose the sensor data for each session into 150 temporally independent components and associated sensor topographies. Artefact components were classified by automated inspection of the spatial topography, time course, kurtosis of the time course and frequency spectrum, and subtracted from the data.

Sequenceness analysis relies on the ability to decode transient spontaneous neural reactivations of task stimulus representations from in MEG data collected during resting-state. First, we characterized participant-specific MEG sensor patterns corresponding to each task picture (A, B, C, …) using visually-evoked MEG patterns from a pre-learning Stimulus Localizer task. As previously described (2), for each task picture (n = 8) we trained a separate one-vs-rest lasso-regularized logistic regression (decoding) model using epoched MEG sensor-level data from Stimulus Localizer, and assessed prediction accuracy

for the family of trained decoders at each time point of the visually-evoked response in leave-one-out cross validation. Group-level cross-validated peak decoding accuracy was at 180 ms after picture onset. We then applied trained decoders (from this peak accuracy time bin) to MEG (sensor-level) data from each time point of the post-learning rest session to generate a [time, state] reactivation probability matrix, and used a Temporally Delayed Linear Modelling (TDLM) framework to quantify evidence for sequential reactivations consistent with the inferred task transition structure (27).

In our previous work (2) we found maximal evidence for spontaneous neural replay at 40 ms *state→state* transition lag (i.e. neural reactivation of state 'A' followed by reactivation of state 'B', 40 ms later). In the present work we therefore identified time points during the rest session where strong reactivation of one stimulus (e.g., A) was followed by strong reactivation of another stimulus that is adjacent in the learned task sequence (e.g., B), with 40 ms lag. As previously described (2), we identified replay events that were preceded by a pre-event baseline of low replay probability, and epoched the post-learning rest MEG data surrounding each such putative replay event. For each epoch (event) we then computed a frequency decomposition (wavelet transformation) in the window -100 to +150 ms with respect to replay onset, for each (non-artefactual) sensor. Averaging this estimate over sensors and epochs resulted in a [time, frequency] matrix for each participant, capturing the typical spectrally-resolved power change at replay onset. As previously reported, this analysis identified a transient increase in high-frequency (ripple band, 120 – 150 Hz) power coinciding with replay onset, compared to a pre-onset baseline (-100 to -10 ms), source localized to hippocampus (2) (**Figure 6A**).

In the present work we extract two MEG-derived variables estimated during post-learning rest session, and correlate these MEG measures with our model-derived semantic association variable ($\Delta\omega$): (1) replay-associated ripple power (i.e., peak power increase in the 120-150 Hz range, occurring 0 – 50 ms (±10 ms) following a replay event, compared to an event free baseline, and measured over all sensors), and (2) replay strength (i.e., mean sequenceness effect detected at 40-50 ms replay lag). These MEG measures are identical to those discussed in detail in our previous published MEG study (2).

In exploratory analyses we also quantified several dynamical 'ripple power' measures in the post-learning MEG rest session. As in our previous work (2), for each

participant we estimated the instantaneous ‘ripple’ power at each time point and channel during the post-learning MEG rest session (using a Hilbert transform on MEG data, sampled at 400 Hz and filtered to 120 – 150 Hz). We defined a ripple ‘event’ as a contiguous stretch of time points each exhibiting ripple power (sum over channels) greater than 2 standard deviations from the subject- and session-specific median (28–30). For each participant, we extracted summary measures characterizing the temporal dynamics of these ripple events, including mean (and SD) ripple ‘duration’ (i.e., number of contiguous suprathreshold timepoints per event) and mean (and SD) ‘interval-time’ (i.e., number of subthreshold timepoints separating two adjacent events) (31).

Statistical analysis and software

Statistical analysis was performed using MATLAB (Mathworks) 2019a. Analysis-specific toolboxes are named in respective sections. Prior to testing group differences or computing correlations using parametric tests (e.g., unpaired t-test, Pearson’s correlation), we conducted a formal test that the effects in question were sampled from a population with a normal distribution (Shapiro Wilk test), and used non-parametric equivalent tests where this null hypothesis was rejected at $\alpha = 0.05$ (e.g., Wilcoxon rank sum test for equal medians, Spearman’s rank correlation). For between-subjects multiple regression analyses, ‘group’ was effects coded (patients = -0.5, controls = +0.5) and the design matrix included the experimental variable under investigation (e.g., $\Delta\omega$), group membership, and the multiplicative interaction of these terms. For all analyses summary effects are reported as mean ± 1 standard error of the mean (SEM), and two-tailed $P < 0.05$ is deemed significant.

For low-dimensional visualization of item sequence trajectories in ‘semantic space’ (**Figure 1** and **Figure 3**) we used the Uniform Manifold Approximation and Projection (UMAP) algorithm (32) implemented in MATLAB (https://www.mathworks.com/matlabcentral/fileexchange/71902). We used cosine distance as the metric in native (300D) space and Euclidean distance as the metric in 2D or 3D space. Embedding vectors for response items with multiple words were defined as the mean vector over individual words. Parameters for 2D projection: minimum distance = 0.2, number neighbors = 25, spread 1.0.

Supplemental Results

List length

PScz generated fewer unique valid words compared to control participants in the ‘category’ fluency task (controls = 54.1 ± SEM 2.70, PScz = 46.9 ± 1.87, z(50) = 2.13, P = 0.03, Wilcoxon rank sum test, two tailed, as in (33–35)), but not in the ‘letter’ fluency task (control = 39.7 ± 2.60, PScz = 41.7 ± 2.90, t(50) = -0.50, P = 0.62, two sample t-test, two tailed) (**Figure 2A**), in line with previous findings (9, 36, 37).

Comparison of semantic distance between consecutive words vs. all unique pairs

Across all participants, median semantic distance was lower between word pairs that occupied adjacent positions in observed lists (e.g., *‘1. Cat’ - ‘2. Dog’*), compared to the distribution of such distances observed for all possible word pairs (e.g., *‘1. Cat’ - ‘9. Fish’*) (‘Category’ task: median adjacent pair distance across all participants = 0.56 [inter-quartile range (IQR) 0.44 – 0.64], all pair distance = 0.64 [0.58 – 0.69], difference between medians P < 0.0001. ‘Letter’ task: adjacent pair distance = 0.67 [0.59 – 0.73], all pair distance = 0.69 [0.64 – 0.74], difference between medians P < 0.0001, Wilcoxon rank sum test of equal medians, two tailed, **Figure 2B**). This semantic influence was more pronounced in the ‘category’ task compared to the ‘letter’ task (P < 0.0001, Wilcoxon rank sum test of equal medians of consecutive word distances, two tailed), suggesting that task demands influence the degree to which semantic information constrains (guides) the search process.

Semantic distance as a function of item number

In the ‘category’ task semantic distance between consecutive words increased as a function of the number of words already produced (indicative of fatigue and/or reduction of remaining words in vocabulary). However, the rate of this increase was not significantly different between groups (mean regression coefficients from regression of semantic distance on item number in ‘category’ task: controls = $8.50*10^{-4} \pm 2.48*10^{-4}$, PScz $1.3*10^{-3} \pm 2.46*10^{-4}$, t(50) = -1.33, P = 0.19). (We similarly found no group difference in the ‘letter’ task.)

Trajectories through semantic communities and analysis of retrieval times

As described in the main text, in the 'category' task, we sought to test whether semantic search trajectories display signatures of being influenced by a meso-scale structure of the cognitive map – namely, whether they are influenced by distinct semantic communities. A prediction of this effect is that trajectories may linger within coherent semantic communities for a period of time, before transitioning to a new community, where such 'community switches' are associated with a measurable time delay (19). In the animal foraging literature such community-based search trajectories are expected under formal accounts of foraging optimality (19, 38), and may arise from biologically plausible sequence generation mechanisms within hippocampal-entorhinal cortex (39).

To investigate this phenomenon in the 'category' task data, we identified nonoverlapping (semantic) communities in the set of unique items generated across all participants by applying a data-driven agglomerative clustering algorithm to the [item, item] semantic similarity matrix (see **Identifying community structure in animal space**). This approach partitioned the set of all items into 5 nonoverlapping communities which correspond to intuitive animal categories with good face validity **Figure 4A**.

To assess predictive validity, we asked whether information pertaining to semantic community assignment was predictive of time intervals between response items ('retrieval times', RT), over and above the predictive effect attributable to semantic distance *per se*. In effect, this analysis asks whether the presence of a transition between communities (e.g., transitioning from 'flying creatures' to 'sea creatures') can predict an increase in RT even after accounting for the local pairwise similarity information from which the community partition solution was derived. For each participant we regressed $\ln(RT)$ onto a design matrix comprising predictor variables for pairwise semantic distance, presence of a community 'switch' (binary variable), a regressor controlling for the effect of fatigue (or exhaustion of items in memory), and a constant term. The community switches predictor explained a significant proportion of unique variance in $\ln(RT)$ across all participants ($z(51) = 4.31$, $P < 0.001$, one sample Wilcoxon sign test for effect different to 0, two-tailed , control mean $\beta_{switch} = 0.25 \pm 0.07$, PScz mean $\beta_{switch} = 0.23 \pm 0.07$), with no difference between groups ($z(50) = -0.06$, $P = 0.95$, Wilcoxon rank sum test, **Figure 4B**). As expected, we also found large and significant predictive effects from (local) semantic similarity

between words (control mean $\beta_{similarity}$ = 3.20 ± 0.28, PScz $\beta_{similarity}$ = 2.88 ± 0.27, t(51) = 15.8, P < 0.001, one sample t-test, two-tailed), and word number expressed as a proportion of list length (a proxy for fatigue or word list depletion, controls $\beta_{fatigue}$ = 1.06 ± 0.09, PScz $\beta_{fatigue}$ = 1.24 ± 0.13, t(51) = 14.4, P < 0.001, one sample t-test, two-tailed), again with no significant group difference ($\beta_{similarity}$ t(50) = 0.84, P = 0.41; $\beta_{fatigue}$ t(50) = -1.11, P = 0.27, two sample t-tests, two tailed). We interpret the lack of any group difference in the $\beta_{similarity}$ coefficient as indicating that the internal semantic representations of both PScz and controls are equally well characterized by the pre-trained semantic word embeddings.

Having assigned each item to exactly one community, we could then investigate the participant-specific search trajectories through communities (**Figure 4**).

We considered a possibility that the reduction in model-derived semantic salience (**Figure 5B**) might be attributable to a tendency for PScz to switch between semantic communities more than control participants. Previous analytic approaches have construed semantic search as a two-step process, in which periods of 'local search' (governed by the association strength between recent response items, as in our modelling approach) are separated by periods of 'global search' (community switch, where semantic similarity plays no role) (8, 9, 19). In light of this, we re-fitted the winning computational model to all participants a second time, now ensuring that 'community switch' response items did not affect the model likelihood estimation. This analysis again showed that PScz exhibit a reduced influence of semantic association compared to controls in the 'category' task ($group * task$ interaction on β_{sem} variable, P = 0.049), with no such effect for the orthographic salience parameter ($group * task$ interaction on β_{orth} variable, P = 0.81).

We refrained from conducting similar community trajectory analyses in the 'letter' task, or in either task using orthographic distance metric, as the community partitions derived from these data and lacked face validity.

Trajectories through orthographic space

We repeated the trajectory analysis (**Figure 3**) using a non-semantic association measure (i.e., orthographic [Levenshtein] distance (15), i.e., letter-by-letter word

similarity, a proxy for phonemic similarity), which we reasoned would exhibit a boosted influence on the word selection process when semantic information is not immediately task relevant (as in the 'letter' task), and where this influence might be similarly expressed in PScz and controls (given an emphasis on semantics in classical descriptions of thought disorder (40), and findings showing largely preserved phonemic similarity in PScz during 'letter' fluency (9)).

Consistent with this hypothesis, the orthographic distance separating consecutive words was significantly smaller in the 'letter' task (control: mean orthographic distance = 0.34 ± 0.009, PScz: mean orthographic distance = 0.36 ± 0.009) compared to the 'category' task (control: mean orthographic distance = 0.48 ± 0.005, PScz: mean orthographic distance = 0.49 ± 0.005), with no group difference (*group * task* ANOVA on orthographic distance: main effect of task: F(50,1) = 305.3, P < 0.001; main effect of group: F(50,1) = 0.004, P = 0.09; *group * task* interaction: F(50,1) = 1.10, P = 0.30). Optimality divergence analyses using orthographic distance, likewise, revealed no significant group differences (**Figure S3**). Intriguingly, while we found no relationship between (orthographic) global optimality divergence and task performance in the 'category' task (r(50) = 0.08, P = 0.59, Pearson's correlation), such a relationship did exist for the 'letter' task (r(50) = -0.30, P = 0.03, Pearson's correlation), and remained significant when controlling for group differences in list length *per se* (*list length ~ group * optimality divergence* multiple regression. $\beta_{optimality\_divergence}$ = -4.48 ± 1.77, t(48) = -2.53, P = 0.015, β_{group} = 14.15 ± 7.30, t(48) = 1.94, P = 0.06. $\beta_{group*optimality\_divergence}$ = -7.06 ± 3.54, t(48) = -1.20, P = 0.05).

Supplemental Figures and Table

Figure S1

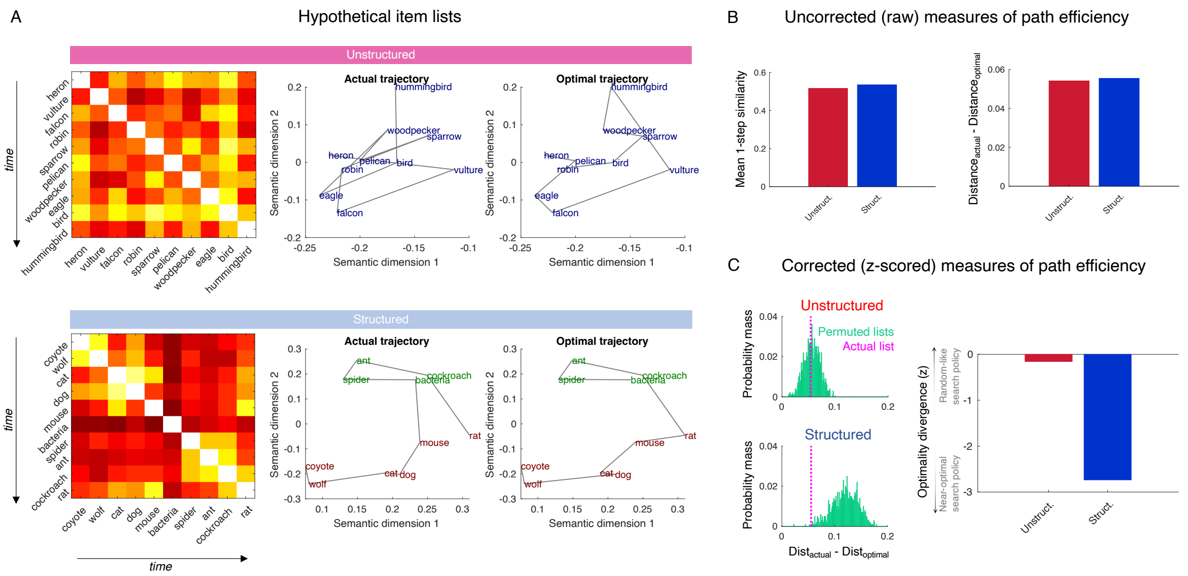

Figure S1: Quantifying efficiency of semantic search paths.
Related to **Figure 3** and **SI Appendix Methods**.
(A) When quantifying path optimality it is important to control for between-subject differences in list membership. Two hypothetical item lists depicted as similarity weight matrices ordered by item position *(left)* and as paths through semantic space *(middle)*. In the extreme case one hypothetical participant may generate a relatively unstructured list of animals belonging to a single semantic cluster (e.g., birds, *top*), while another *(bottom)* may generate a more diverse list of exemplars in a more structured manner (indicated by block diagonal structure in semantic similarity weight matrix). The optimal path (i.e., the trajectory visiting each item exactly once, minimizing distance travelled) is depicted on the right (item color randomly assigned to community allocation derived from an agglomerative clustering algorithm on the similarity weight matrix of all response items).
(B) Measures of path optimality that do not correct for between-subject differences in list membership (*left*: mean cosine similarity of consecutive pairs, *right*: difference in mean distance per edge between optimal and actual paths, i.e., $distance_{actual}$ - $distance_{optimal}$) are unable to identify path optimality differences between participants.
(C) However, by comparing summary effects to a null distribution generated from participant-specific random list shuffles, it is evident that one participant exhibits a semantic trajectory that is highly non-random with respect to semantic information. *Left:* Raw $distance_{actual}$ - $distance_{optimal}$ effect (magenta) plotted alongside the distribution of the effect under 1000 random list permutations (green). *Right:* Optimality divergence, defined as z-score of $distance_{actual}$ - $distance_{optimal}$ effect vs. permuted distribution. Of note, the optimality divergence z-score is mathematically identical to the cosine distance traversed per step (1-simiarity, in B *left*), expressed as a z-score vs. the null distribution expected under random list shuffles.

Figure S2

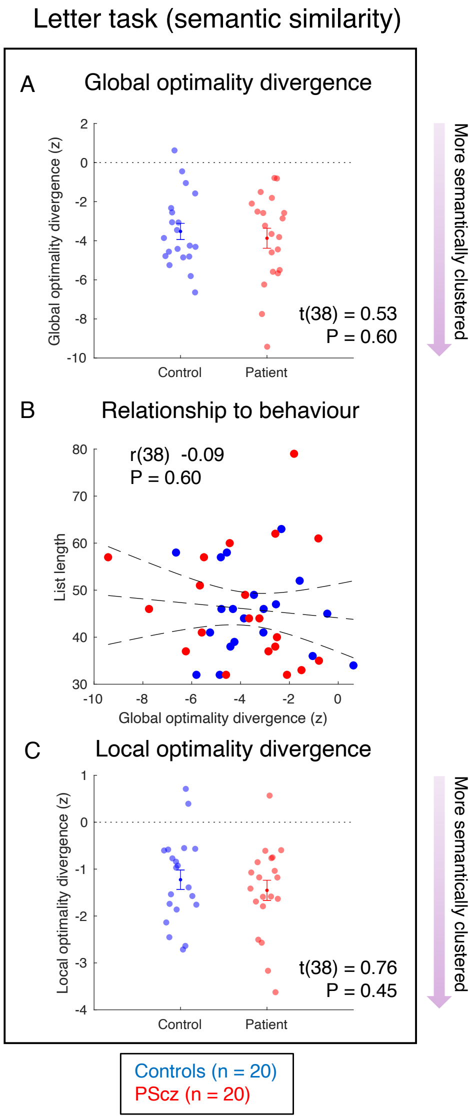

Figure S2: Semantic path optimality analysis in 'letter' task (using window length = 32). Related to **Figure 3.**

(A) Global optimality divergence (*distance$_{actual}$ – distance$_{optimal}$*) expressed as a z-score against participant-specific list shuffles such that 0 indicates a near-random word selection process (random sampling policy), and increasingly negative values indicate an optimality divergence that is smaller (i.e., improved) with respect to the random word selection process. Semantic distance used.

(B) Relationship between global optimality divergence (expressed as the z-score of optimality divergence vs. a random word selection process) and total items retrieved from memory in 5 minutes. Pearson's correlation, 2 tailed.

(C) Local optimality divergence, as in **Figure 3**. Two sample t test, two tailed.

Mean ± SM over participants. n = 40 (20 PScz and 20 controls) as 12 participants were excluded for having a list length shorter than the window length of 32.

Figure S3

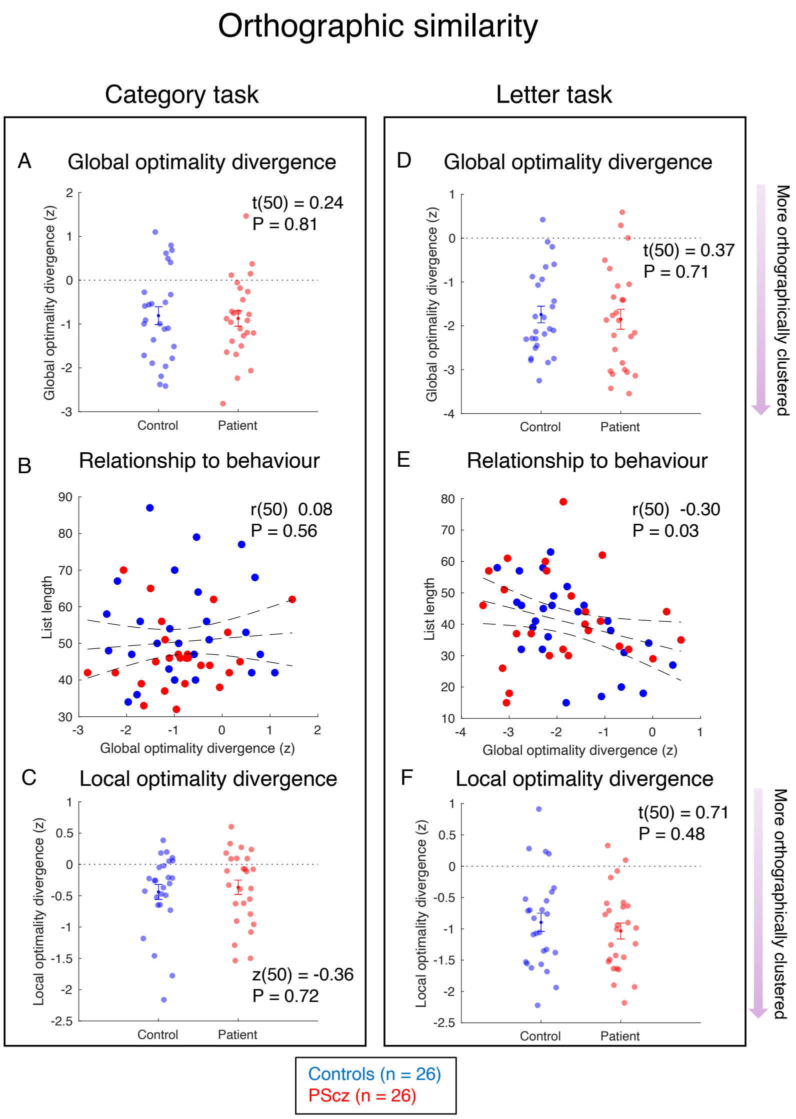

Figure S3: Orthographic path optimality analysis. Related to **Figure 3.**

(A) Global optimality divergence (*$distance_{actual}$ – $distance_{optimal}$*) expressed as a z-score against participant-specific list shuffles such that 0 indicates a near-random word selection process (random sampling policy), and increasingly negative values indicate an optimality divergence that is smaller (i.e., improved) with respect to the random word selection process. Distance metric used is orthographic distance. Two sample t-test, two tailed.

(B)) Relationship between global optimality divergence (expressed as the z-score of optimality divergence vs. a random word selection process) and total items retrieved from memory in 5 minutes. Pearson's correlation, 2-tailed.

(C) Local optimality divergence, defined as in **Figure 3**. Wilcoxon rank sum test, two tailed.

(D) As (A) but using data from the 'letter' task. Two sample t-test, two tailed.

(E) As (B) but using data from the 'letter' task. Pearson's correlation.

(D) As (C) but using data from the 'letter' task. Two sample t-test, two tailed.

Group comparisons (A, C, D & F) expressed as mean ± SEM. Error bars on linear trend lines (B & E) reflect 95% confidence intervals of the linear regression slope. Window length for all analysis equals the task-specific minimum list length across all participants (for 'category' task = 32, for 'letter' task = 15 [similar results using window length of 32]). Sample n = 26 controls, n = 26 PScz.

Figure S4

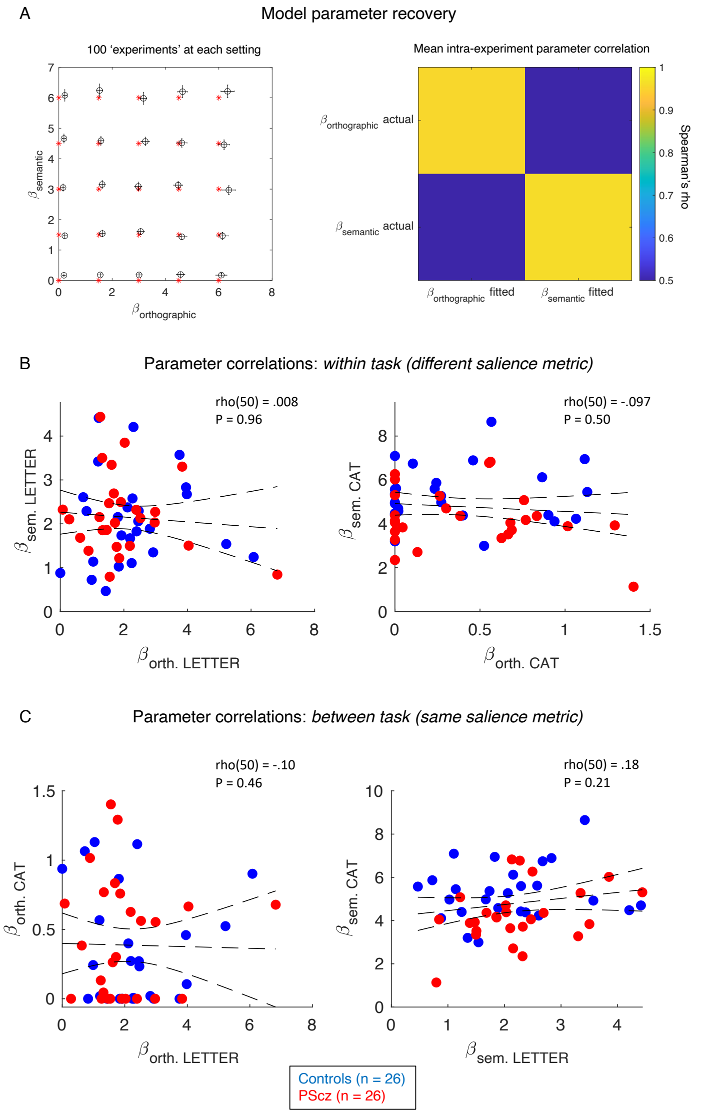

Figure S4: Parameter recovery and correlations of winning computational model.
Related to **Figure 5.**

(A) Parameter recovery for the winning arbitration model, containing 2 free parameters for each task (**Equation 10**, i.e., 4 free parameters when fitting to data concatenated over 2 tasks). *(Left)*: ground truth parameter setting (red) and recovered parameters (mean ± 2 SEM over 100 experiments, see SI Appendix Methods for details). *(Right)*: For each experiment we calculated the Spearman's rank correlation (color bar) between both generative parameters and both recovered parameters, and display the mean over these correlation coefficients.

(B) For each participant we fit a separate semantic and orthographic salience parameter for each task (letter, *left*, and category, *right*), which compete to explain the task-specific behavioral data. There is no correlation between these parameters ('within task') across participants, in line with the notion that they explain separate sources of variance in the data.

(C) We also find no correlation (across participants) in the rank order of the orthographic *(left)* or semantic *(right)* salience parameters across tasks (independently estimated in the winning model).

Parameters in (B) and (C) from the winning computational model (4P model, see **Figure 5** & **Equation 10**). Error bars indicate 95% confidence interval on the linear line of best fit. Correlation coefficients and P values from Spearman's rank correlation. Sample: PScz n = 26, controls n = 26.

Figure S5

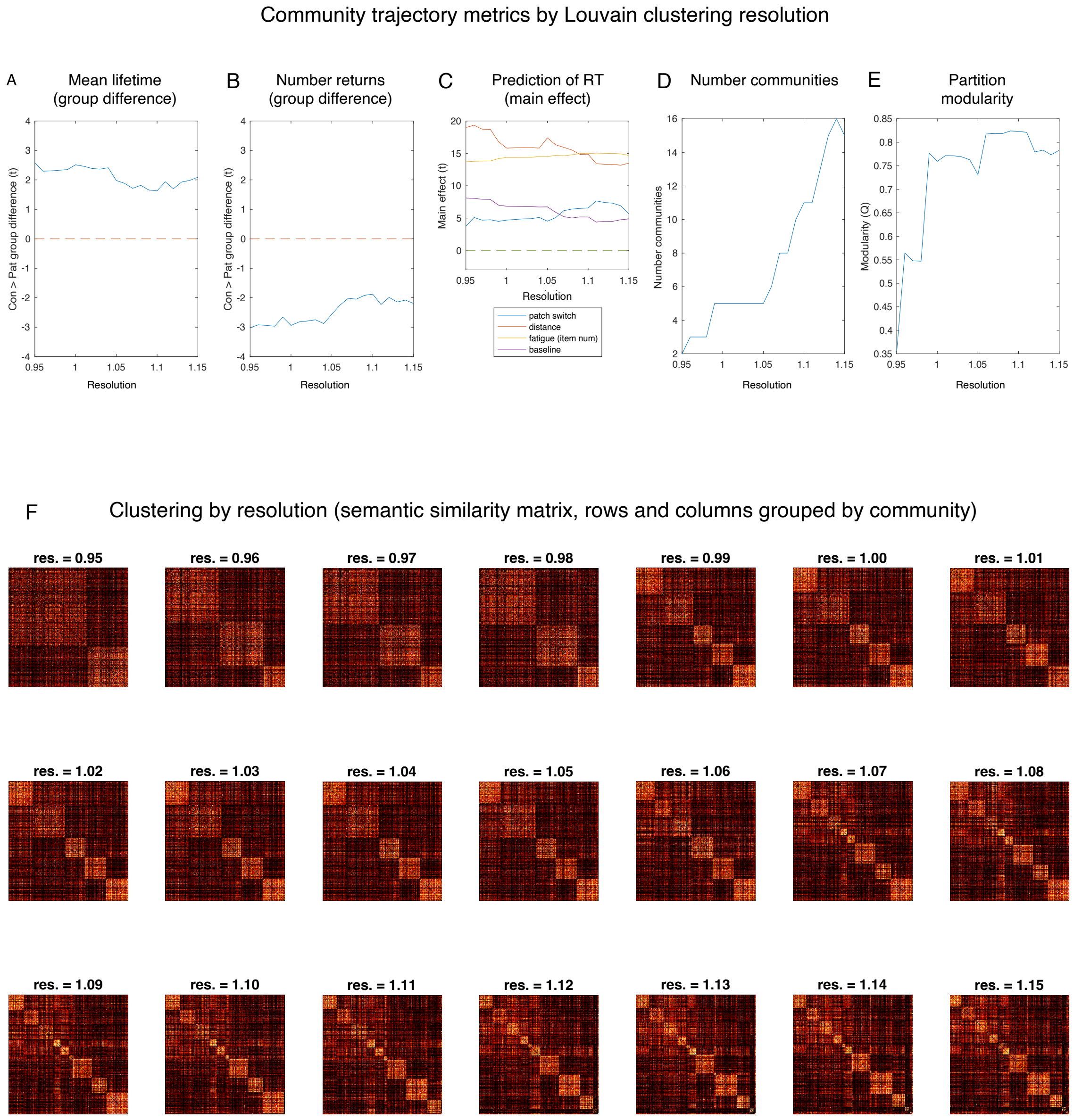

Figure S5: Effect of clustering algorithm resolution parameter on reported community trajectory effect sizes (‘category’ task, semantic similarity).
Related to **Figure 4.**
(A) We repeated the clustering analysis separately over a range of resolution parameters ($\lambda = [0.95: 0.01: 1.15]$), and repeated the community-based trajectory and retrieval time (RT) analysis in an identical manner to the main results. In (A) we show the t value of the group difference (two sample t-test) in community lifetimes, plotted as a function of resolution. Positive values indicate that control participants exhibit longer community lifetime effects compared to PScz (effect expressed as a z-score relative to the participant-specific null distribution).
(B) T value of the group difference (two sample t-test) in mean number of return visits to an already-visited community, as a function of resolution. Negative values indicate that control participants exhibit a lower

number of repeat visits compared to PScz (effect expressed as a z-score relative to the participant-specific null distribution).
(C) At each analyzed clustering resolution, and for each participant, we regressed the inter-word retrieval time ($\ln RT$) onto a design matrix comprising the response-specific semantic distance (to preceding item), response number (as a proportion of list length, a proxy for fatigue or memory depletion), a patch switch binary variable (as defined by the resolution-specific partition), and a constant (baseline) term. Here we plot the t-values of the regression coefficients associated with each predictor, taken from a one sample t-test over all participants, as a function of resolution.
(D) Number of communities identified at each resolution.
(E) Graph modularity (**Equation 12)** of the final partition solution at each resolution.
(F) Similarity matrices, rows and columns ordered so as to group items belonging to the same clusters (block diagonal structure), such that the ordering is not consistent between panels.
For trajectory analyses (A and B) window length = 33 (minimum list length over all participants). Consecutive repetitions removed from participant lists (non-consecutive repetitions remain). Sample: controls n = 26, PScz n = 26. All analyses conducted on the 'category' task word lists, using semantic distance.

Table S1

Variable	Healthy volunteers	Patients	Group comparison[#]
**Demographic**			
Sample size	27	26	
Gender	6 F, 21 M	6 F, 20 M	x^2 = 0.006 (P = 0.95)
Age (mean, SEM)	27.7 (6.1)	28.5 (5.2)	z = -0.76 (P = 0.45)
Years in education (mean, SD)	18.2 (3.5)	17.8 (2.6)	t = 0.53 (P = 0.60)
Employment status [F/P/S/U]*	7 / 7 / 8 / 5	9 / 3 / 3 / 11	x^2 = 6.36 (P = 0.10)
Handedness	23R, 4L	24R, 2L	x^2 = 0.67 (P = 0.41)
Ethnicity [W / BAME / Other][†]	10 / 15 / 2	8 / 16 / 2	x^2 = 0.24 (P = 0.89)
Alcohol units week^{-1} (mean, SD)	3.74 (5.6)	3.58 (6.4)	z = 0.85 (P = 0.40)
Recreational cannabis (not within 1 week)	9	9	x^2 = 0.01 (P = 0.92)
Current smoker (not within 6 hours)	6	13	x^2 = 4.44 (P = 0.04)
**Cognitive**			
IQ (SD)	104.0 (5.6)	105.2 (7.9)	z = -1.11 (P = 0.27)
Digit span forward (mean, SD)	6.34 (0.92)	6.29 (1.11)	z = 0.41 (P = 0. 68)
Digit span backward (mean, SD)	4.17 (0.99)	3.87 (1.11)	z = 1.12 (P = 0.26)
**Psychiatric symptoms and signs**			
Depressive symptoms[‡] (mean, SD)	0.93 (2.53)	9.35 (8.02)	z = -5.57 (P < 0.001)
Positive psychotic symptoms[§] (mean, SD)	7.15 (0.36)	14.4 (6.45)	z = -5.69 (P < 0.001)
Negative psychotic symptoms[§] (mean, SD)	7.07 (0.27)	13.6 (6.38)	z = -5.86 (P < 0.001)
General psychopathology[§] (mean, SD)	16.4 (1.01)	25.5 (7.67)	z = -6.20 (P < 0.001)
General assessment functioning[‖] (mean, SD)	98.0 (5.4)	68.6 (15.2)	z = 6.22 (P < 0.001)
**Clinical Details**			
Num. taking D2/3R antagonist medication	-	13[¶]	-
Months since symptom onset (median, IQR)	-	45 (30 - 60)	-
Num. psychotic episodes (median, IQR)	-	3 (2 - 4)	-
Num. inpatient admissions (median, IQR)	-	1 (1 - 4)	-

Table S1. Participant demographic, cognitive and clinical information.
Related to Methods.

[*] F = fulltime employment, P = part-time employment, S = student, U = unemployed.

[†] W = White. BAME = Black, Asian, and Minority Ethnic. Other includes multiple ethnic groups.

[‡] Montgomery Åsberg Depression Rating Scale (MADRS), floor = 0.

[§] Positive and Negative Syndrome Scale (PANSS) scale, floor = 7(positive), 7(negative), 16(general).

[‖] General Assessment of Functioning (GAF) scored from 0 – 100.

[¶] D2/3 antagonist medication per medicated patient: (1) olanzapine 15 mg day^{-1}, (2) olanzapine 10 mg day^{-1}, (3) lurasidone 37 mg day^{-1}, (4) risperidone 3 mg day^{-1}, (5) aripiprazole 400mg month^{-1} (depot), (6) risperidone 0.5 mg day^{-1}, (7) aripiprazole 5 mg day^{-1}, (8) olanzapine 7.5 mg day^{-1}, (9) olanzapine 10 mg day^{-1}, (10) amisulpride 400 mg day^{-1} & aripiprazole 5 mg day^{-1}, (11) paliperidone 50 mg month^{-1} (depot), (12) paliperidone 175 mg 3-month^{-1} (depot), (13) paliperidone 50 mg month^{-1} (depot).

[#]Group comparisons: unpaired t-test or Wilcoxon rank sum test for continuous variables (normally and non-normally distributed, respectively), Chi squared test for categorical variables. All P values are two-tailed.
SD: standard deviation. IQR: inter-quartile range.

SI References

1. M. B. First, R. L. Spitzer, M. Gibbon, J. B. W. Williams, *Structured Clinical Interview for DSM-IV Axis I disorders— Patient Edition*, Version 2 (New York Biometrics Research Department, 1995).

2. M. M. Nour, Y. Liu, A. Arumuham, Z. Kurth-Nelson, R. J. Dolan, Impaired neural replay of inferred relationships in schizophrenia. *Cell* **184** (2021).

3. S. Kay, A. Fiszbein, L. Opler, The Positive and Negative Syndrome Scale (PANSS) for schizophrenia. *Schizophr Bull.* **13**, 261–276 (1987).

4. J. B. W. Williams, K. A. Kobak, Development and reliability of a structured interview guide for the Montgomery-Åsberg Depression Rating Scale (SIGMA). *British Journal of Psychiatry* **192**, 52–58 (2008).

5. American Psychiatric Association, *Diagnostic and Statistical Manual of Mental Disorders*, 5th Ed. (American Psychiatric Publishing, 2013).

6. D. Wechsler, *Wechsler test of adult reading: WTAR* (The Psychological Corporation, 2001).

7. B. Elvevåg, P. W. Foltz, D. R. Weinberger, T. E. Goldberg, Quantifying incoherence in speech: An automated methodology and novel application to schizophrenia. *Schizophrenia Research* **93**, 304–316 (2007).

8. N. B. Lundin, *et al.*, Semantic Search in Psychosis: Modeling Local Exploitation and Global Exploration. *Schizophrenia Bulletin Open* **1**, 1–11 (2020).

9. N. B. Lundin, M. N. Jones, E. J. Myers, A. Breier, K. S. Minor, Semantic and phonetic similarity of verbal fluency responses in early-stage psychosis. *Psychiatry Research* **309** (2022).

10. E. A. Solomon, B. C. Lega, M. R. Sperling, M. J. Kahana, Hippocampal theta codes for distances in semantic and temporal spaces. *Proceedings of the National Academy of Sciences*, 611681 (2019).

11. T. Mikolov, E. Grave, P. Bojanowski, C. Puhrsch, A. Joulin, Advances in pre-training distributed word representations. *LREC 2018 - 11th International Conference on Language Resources and Evaluation*, 52–55 (2018).

12. H. Lane, H. Cole, H. M. Hapke, *Natural language processing in action*, 1st Ed. (Manning Publications Co., 2019).

13. S. T. Piantadosi, F. Hill, Meaning without reference in large language models. *arXiv* (2022) https:/doi.org/https://doi.org/10.48550/arXiv.2208.02957 (December 17, 2022).

14. T. Mikolov, K. Chen, G. Corrado, J. Dean, Efficient estimation of word representations in vector space. *1st International Conference on Learning Representations, ICLR 2013 - Workshop Track Proceedings*, 1–12 (2013).

15. V. I. Levenshtein, Binary codes capable of correcting deletions, insertions, and reversals. *Soviet Physics Doklady* **10**, 707–710 (1966).

16. C. R. Nettekoven, *et al.*, Semantic Speech Networks Linked to Formal Thought Disorder in Early Psychosis. *Schizophrenia Bulletin* **49**, S142–S152 (2023).

17. L. Palaniyappan, *et al.*, Speech structure links the neural and socio-behavioural correlates of psychotic disorders. *Progress in Neuro-Psychopharmacology and Biological Psychiatry* **88**, 112–120 (2019).

18. T. T. Hills, P. M. Todd, M. N. Jones, Foraging in Semantic Fields: How We Search Through Memory. *Topics in Cognitive Science* **7**, 513–534 (2015).

19. T. T. Hills, M. N. Jones, P. M. Todd, Optimal foraging in semantic memory. *Psychological Review* **119**, 431–440 (2012).

20. V. D. Blondel, J. L. Guillaume, R. Lambiotte, E. Lefebvre, Fast unfolding of communities in large networks. *Journal of Statistical Mechanics: Theory and Experiment*, 1–12 (2008).

21. A. Lancichinetti, S. Fortunato, Consensus clustering in complex networks. *Scientific Reports* **2** (2012).

22. M. Rubinov, O. Sporns, Complex network measures of brain connectivity: Uses and interpretations. *NeuroImage* **52**, 1059–1069 (2010).

23. A. Fornito, A. Zalensky, E. Bullmore, *Fundamentals of Brain Network analysis* (Academic Press, 2016).

24. J. R. Cohen, M. D'Esposito, The Segregation and Integration of Distinct Brain Networks and Their Relationship to Cognition. *Journal of Neuroscience* **36**, 12083–12094 (2016).

25. M. M. Nour, *et al.*, Task-induced functional brain connectivity mediates the relationship between striatal D2 / 3 receptors and working memory. *eLife* **8**, 1–23 (2019).

26. Y. Liu, R. J. Dolan, Z. Kurth-Nelson, T. E. J. Behrens, Human Replay Spontaneously Reorganizes Experience. *Cell* **178**, 640–652 (2019).

27. Y. Liu, *et al.*, Temporally delayed linear modelling (TDLM) measures replay in both animals and humans. *eLife* **10**, e66917 (2021).

28. C. G. McNamara, Á. Tejero-Cantero, S. Trouche, N. Campo-Urriza, D. Dupret, Dopaminergic neurons promote hippocampal reactivation and spatial memory persistence. *Nature Neuroscience* **17**, 1658–1660 (2014).

29. G. M. van de Ven, S. Trouche, C. G. McNamara, K. Allen, D. Dupret, Hippocampal Offline Reactivation Consolidates Recently Formed Cell Assembly Patterns during Sharp Wave-Ripples. *Neuron* **92**, 968–974 (2016).

30. A. K. Gillespie, *et al.*, Hippocampal replay reflects specific past experiences rather than a plan for subsequent choice. *bioArXiv* (2021).

31. C. Higgins, *et al.*, Replay bursts in humans coincide with activation of the default mode and parietal alpha networks. *Neuron*, 1–12 (2021).

32. L. McInnes, J. Healy, J. Melville, UMAP: Uniform Manifold Approximation and Projection for Dimension Reduction (2020) (June 16, 2023).

33. J. Egeland, T. L. Holmen, G. Bang-Kittilsen, T. T. Bigseth, J. A. Engh, Category fluency in schizophrenia: opposing effects of negative and positive symptoms? *Cognitive Neuropsychiatry* **23**, 28–42 (2018).

34. K. K. Nicodemus, *et al.*, Category fluency, latent semantic analysis and schizophrenia: A candidate gene approach. *Cortex* **55**, 182–191 (2014).

35. L. Pauselli, *et al.*, Computational linguistic analysis applied to a semantic fluency task to measure derailment and tangentiality in schizophrenia. *Psychiatry Research* **263**, 74–79 (2018).

36. C. E. Bokat, T. E. Goldberg, Letter and category fluency in schizophrenic patients: a meta-analysis. *Schizophrenia Research* **64**, 73–78 (2003).

37. R. I. Mesholam-Gately, A. J. Giuliano, S. V. Faraone, K. P. Goff, L. J. Seidman, Neurocognition in First-Episode Schizophrenia: A Meta-Analytic Review. *Neuropsychology* **23**, 315–336 (2009).

38. E. Charnov, Optimal foraging, the marginal value theorem. *Theoretical population biology* **9**, 129–136 (1976).

39. D. C. McNamee, K. L. Stachenfeld, M. M. Botvinick, S. J. Gershman, Flexible modulation of sequence generation in the entorhinal–hippocampal system. *Nature Neuroscience* (2021) https:/doi.org/10.1038/s41593-021-00831-7.

40. P. Casey, B. Kelly, *Fish's Clinical Psychopathology: Signs and Symptoms in Psychiatry* (2007).
